# Ecogeographic Study of *Ipomoea* Species in Mauritius, Indian Ocean

**DOI:** 10.3390/plants13192706

**Published:** 2024-09-27

**Authors:** Yakshini Boyjnath, Mohammad Ehsan Dulloo, Vishwakalyan Bhoyroo, Vijayanti Mala Ranghoo-Sanmukhiya

**Affiliations:** 1 Department of Agricultural and Food Science, Faculty of Agriculture, University of Mauritius, Réduit 80837, Mauritius; v.bhoyroo@uom.ac.mu (V.B.); m.sanmukhiya@uom.ac.mu (V.M.R.-S.); 2 Alliance of Bioversity International and CIAT, Rose-Hill 71368, Mauritius; e.dulloo@cgiar.org

**Keywords:** conservation, ecogeographical analysis, ecology, distribution, *Ipomoea*, crop wild relatives, invasive species

## Abstract

The wild relatives of crops play a critical role in enhancing agricultural resilience and sustainability by contributing valuable traits for crop improvement. Shifts in climatic conditions and human activities threaten plant genetic resources for food and agriculture (PGRFA), jeopardizing contributions to future food production and security. Studies and inventories of the extant agrobiodiversity, in terms of numbers and distribution patterns of species and their genetic diversity, are primordial for developing effective and comprehensive conservation strategies. We conducted an ecogeographic study on *Ipomoea* species and assessed their diversity, distribution, and ecological preferences across different topographic, altitudinal, geographical, and climatic gradients, at a total of 450 sites across Mauritius. Species distribution maps overlaid with climatic data highlighted specific ecological distribution. Principal Component Analysis (PCA) revealed species distribution was influenced by geographical factors. Regional richness analyses indicated varying densities, with some species exhibiting localized distributions and specific ecological preferences while the other species showed diverse distribution patterns. Field surveys identified 14 species and 2 subspecies out of 21 species and 2 subspecies of *Ipomoea* reported in Mauritius. A gap in ex situ germplasm collections was observed and several species were identified as threatened. Further investigations and a more long-term monitoring effort to better guide conservation decisions are proposed.

## 1. Introduction

Agrobiodiversity the local varieties and breeds of domesticated plants and animals and their wild relatives are rapidly disappearing from our landscapes as evidenced by the most recent global assessment reports on Biodiversity and Ecosystem System [1] and State of the World’s Biodiversity for Food and Agriculture [2]. The loss of the genetic diversity of cultivated plants and local breeds, and the lack of conservation actions to safeguard them, severely undermines the resilience of many agricultural systems to threats such as pests, pathogens, and climate change and poses a serious risk to global food production and security [1]. Furthermore, agrobiodiversity constitutes reservoirs of useful genes and gene complexes that provide insurance for future breeding and adaptation [3,4]. Their conservation is vital to conserving the genetic diversity of landraces, breeds, and wild relatives for current and future breeding programs [5].

It is a fact that we do not have enough information about the extent of diversity of specific crops on farms, in natural ecosystems, and in gene banks, as monitoring systems for agrobiodiversity are lacking at the global, regional, and national levels [2,3,6]. There exists a notable gap in the genetic diversity of vital crop gene pools within ex situ germplasm collections [7]. Studies have underscored the alarming need to identify and successfully conserve the genetic diversity present in crop wild relatives (CWRs) [7,8,9] and crop landrace [10,11]. Undertaking surveys and inventories of the extent of agrobiodiversity, in terms of numbers and distribution patterns of species, varieties, and breeds, and their genetic diversity, is thus primordial for developing effective and comprehensive conservation strategies [12,13,14,15]. Such strategies rely heavily on multidisciplinary datasets [15], often referred to as “agroecological” [16,17] or “bioclimatic” data [17,18].

Ecogeographic studies play a pivotal role in gathering acquiring and analyzing those in-depth multivariate datasets [12,14,15,19,20]. In recent years, there have been major advances in the ecogeographical approaches. Building upon the foundational framework proposed by Maxted et al. [21], the ecogeographic model [14] now encompasses additional dimensions, including ecogeographical diversity assessment, taxonomic diversity assessment [22], genetic diversity assessment [22,23,24], threat assessment [15,22], GIS analysis [15,25] and prediction and climate change impact assessment [15]. All data scientifically combined enable the understanding of geographic distribution patterns, and affiliations between the ecological factors and the survival of a particular species [15,21,26].

With the rising importance of CWRs and their conservation, ecogeographic surveys and studies have been applied to a wide range of crop and wild species such as Coffea [27], Phaseolus beans [15], Spanish common bean landraces [17], Lebanese species [28], and *Ipomoea* species [29]. Khoury et al. [30] carried out a potential distribution model for the fourteen (14) CWRs species of *Ipomoea batatas* (L.) Lam., and assessed the comprehensiveness of ex situ collections of sweetpotato CWRs. 

The Convolvulaceae family, comprising approximately 1600 species across 57 genera [31], is represented in Mauritius by 10 genera and 39 species [32]. Within this family, *Merremia peltata* (L.) Merr. is the only native species in Mauritius [33]. *Ipomoea* is a prominent genus within Convolvulaceae [31]. The genus Ipomoea comprises around 800 species [29]. Members of this genus are widely distributed [34,35]. They are predominantly found in tropical and subtropical areas [29,36,37]. However, the center of origin and diversity of many *Ipomoea* species including the domesticated sweetpotato, *I. batatas,* and its wild relatives is from north-western South America and regions of Central America [38,39,40,41,42]. *I. batatas* dispersal from its center of origin towards Europe took place after 1492 and reached African countries in the 1500s [43,44,45]. Sweetpotato was first introduced in Mauritius during the Dutch period (1598–1710), more specifically, in 1669. The occurrences of other *Ipomoea* species were mentioned as from the mid-1700s [46]. Markedly, human-mediated plant translocation is associated with benefits that the plants can impart [47,48,49], and the diversity of *Ipomoea* species introduced in Mauritius has greatly served as food and medicines and used in religious rituals, or for ornamental purposes in early history [37,46,49,50,51]. However, with time, many *Ipomoea* species became naturalized [46], and several of them, such as *I. herderifolia* L., *I. cairica* (L.) Sweet, and *I. nil* (L.) Roth, are considered as weedy invasive plants [49,52,53,54].

*Ipomoea batatas*, exceptionally, is widely cultivated and consumed almost throughout the world, in around 117 countries [55,56]. On a global basis, sweetpotato occupies the seventh place in the world staple production with 7,248,381 hectares of areas harvested and a global production of 86,410,354.75 tons [57]. Asia is the leading producer, contributing 61% of the total output with China alone producing 46,828,761.12 tons [57]. Africa is now ranked as the second largest producer [58], accounting for 32.2% of the world’s total production [57]. Similarly, sweetpotato has gained prominence in several Indian states [59,60], as well as in the United States [61,62]. In Mauritius, sweetpotato is largely cultivated and is considered an important food source [32]. Recent decade data (2013–2022), from Statistics Mauritius [63], indicate that the area under production and the annual sweetpotato production in Mauritius have averaged around 63.60 (±31.24) hectares and 830.28 (±491.58) tonnes, respectively. The production area, production, and per capita consumption trends of sweetpotato have shown fluctuations over the analyzed period, but a drastic increase has been observed for 2021–2022. The area under production and overall production tripled in 2021. 

The present study focuses on conducting a thorough ecogeographic study of wild and cultivated *Ipomoea* on the island of Mauritius, where 21 species and 2 subspecies of *Ipomoea*, including *Ipomoea batatas* have been reported [32]. The objectives included a compilation of detailed information and understanding of the *Ipomoea* species with emphasis on, but not limited to, their distribution, habitat preference, abundance, threat, conservation status, existing conservation gaps, and need for conservation.

## 2. Results

### 2.1. Inventory of Ipomoea Species

A total of 21 species (*I. alba* L., *I. aquatica*, *I. batatas*, *I. cairica*, *I. fimbriosepala Choisy*, *I. hederifolia*, *I. horsfalliae Hook*., *I. indica* (Burm.) *Merr*., *I. littoralis Blume*., *I. mauritiana Jacq*., *I. nil* (L.) *Roth*, *I. obscura* (L.) *Ker Gawl*., *I. ochracea* (Lindl.) *G. Don*, *Ipomoea pes-tigridis* L., *I. purpurea* (L.) *Roth*., *I. quamoclit* L., *I. rubens Choisy*, *I. tilliacea*, *I. triloba*, *I. venosa* (Desr.) *Roem*. & *Schult*., *I. violaceae* L., and 2 subspecies (*I. carnea* subsp. *fistulosa* (Mart. ex Choisy) D.F. Austin, *I. pes-caprae* (L.) R.Br. subsp. *brasiliensis* (L.) Ooststr. were inventoried based on the literature review, especially from the Flore des Mascareignes [32] and through the consultation of herbaria data, including the National Herbarium of Mauritius. The National Herbarium of Mauritius has good holotype representations of 15 species and 2 subspecies of Ipomoea species. Data were collected from the Ipomoea herbaria sheets of these 15 species, viz., *I. alba*, *I. aquatica*, *I. batatas*, *I. cairica*, *I. hederifolia*, *I. horsfalliae*, *I. indica*, *I. mauritiana*, *I. nil*, *I. obscura*, *I. quamoclit*, *I. tilliacea*, *I. triloba*, *I. violaceae* and 2 subspecies: *I. carnea* subsp. *fistulosa*, and *I. pes-caprae* subsp. *brasiliensis* (L.) *Ooststr*.

An inventory of the *Ipomoea* collection at the National Field Gene Bank, located at Nouvelle Découverte in Mauritius showed that there are 62 live accessions of *I. batatas. Ipomoea batatas* has 14 closely related CWRs [30,64,65] and three of them, *Ipomoea littoralis Blume*, *I. tiliacea (Willd.) Choisy*, and *I. triloba* L., occur in Mauritius [32] but the National Field Gene Bank did not hold any accessions of the wild relatives. Further, no passport information on the sweetpotato accessions was available.

### 2.2. Field Study

Among the 21 species and 2 subspecies, 14 species and 2 subspecies were found during the fieldwork. A total of 473 individuals of *Ipomoea* were encountered at 450 sites. These include *I. alba*, *I. aquatic*, *I. batatas*, *I. cairica*, *I. herderifolia*, *I. indica*, *I. littoralis*, *I. nil*, *I. obscura*, *I. ochracea*, *I. purpurea*, *I. quamoclit*, *I. tilliacea*, and *I. triloba*. The 2 subspecies included *I. carnea* subsp. *fistulosa*, and *I. pes-caprae* subsp. *brasiliensis* (L.) Ooststr. Six species including *I. fimbriosepala*, *I. horsfalliae*, *I. mauritiana*, *I. rubens*, *I. pes-tigridis*, *I. venosa,* and *I. violaceae* were not encountered.

**Figure 1:**
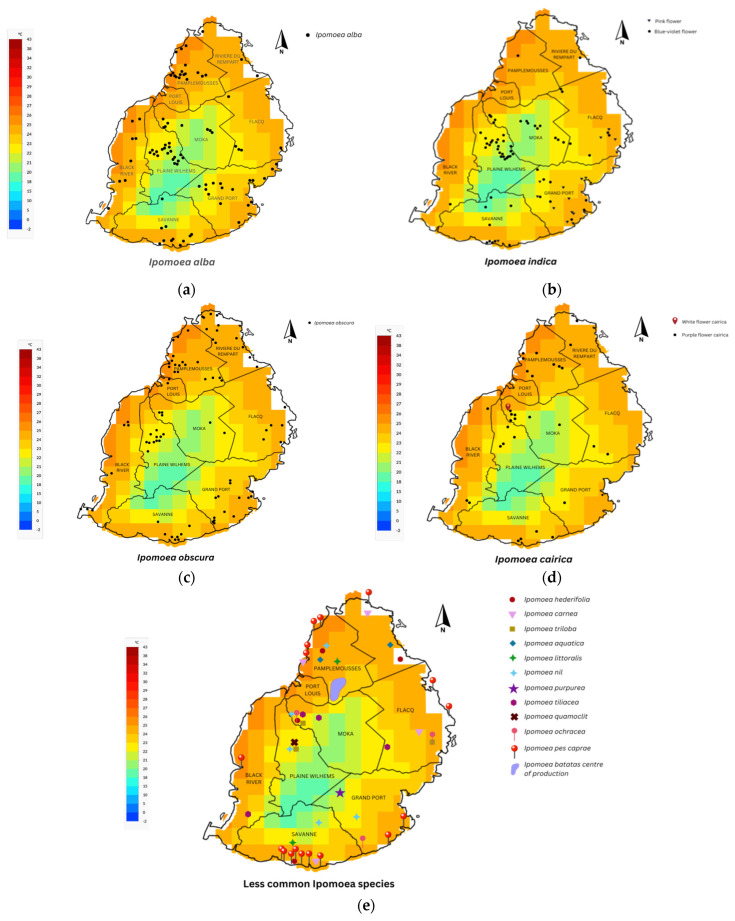
Maps of Mauritius demonstrating extant distribution of *Ipomoea* species: (**a**) *I. alba,* (**b**) *I. indica,* (**c**) *I. obscura,* (**d**) *I. cairica*, and (**e**) other rare species, around Mauritius based on the temperature dispersion over the island.

The island-wide survey showed that there are two main cultivated species of *Ipomoea*, namely, *I. batatas* and *I. aquatica. I. batatas* is mainly cultivated in four northern areas of the island, namely, Montagne Longue, Les Mariannes, Congomah, and Crève-Cœur. These areas serve as the main hub of sweetpotato cultivation and supply sources in Mauritius (see Figure 1e). These regions likely harbor significant diversity and/or landraces of sweetpotato. Otherwise, during the fieldwork, the species were seen as a backyard crop, sometimes in unoccupied farmland, and also as an escape garden in the wild. Different types of sweetpotato were found during this survey that represent the different putative varieties of sweetpotato, which is a subject of a separate study. The reddish-purple skin and white-cream flesh sweetpotato was commonly found at the market. Little Leaf and Raisin are the two common commercial varieties of sweetpotato. Two different types of *I. aquatica* could be distinguished, namely, green type and red type, by the farmers interviewed.

### 2.3. Distribution Patterns of Ipomoea Species

**Figure 2:**
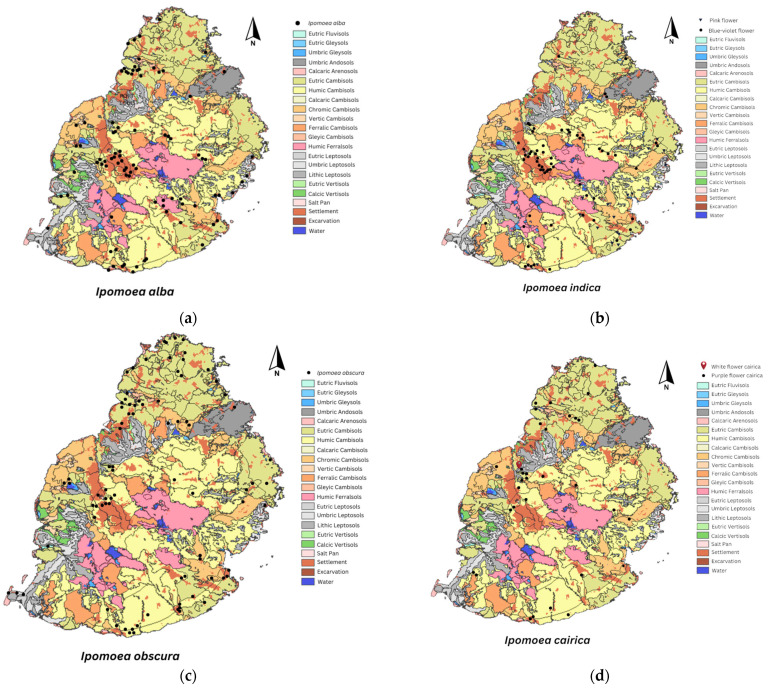

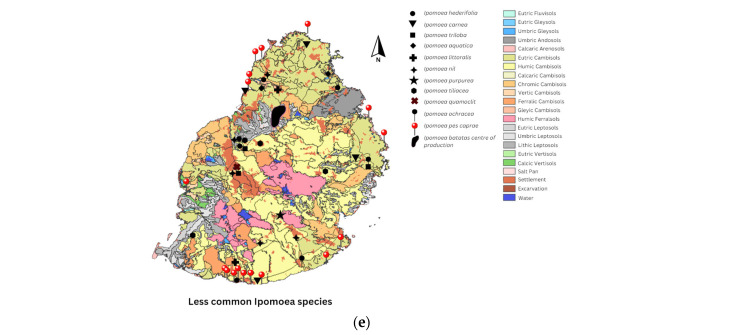
Distribution of (**a**) *I. alba*, (**b**) *I. indica*, (**c**) *I. obscura*, (**d**) *I. cairica*, and (**e**) other rare species, among different soil types.

**Figure 3:**
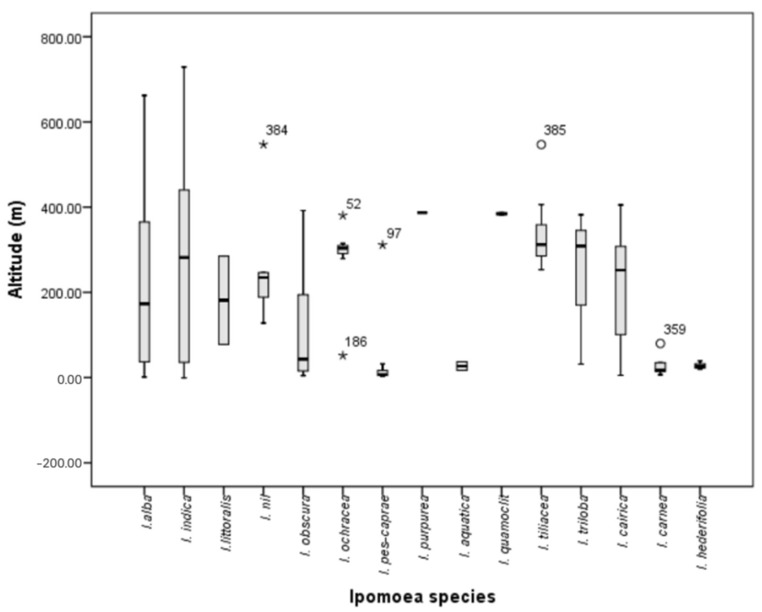
Altitudinal variation of *Ipomoea* by taxa.

**Figure 4:**
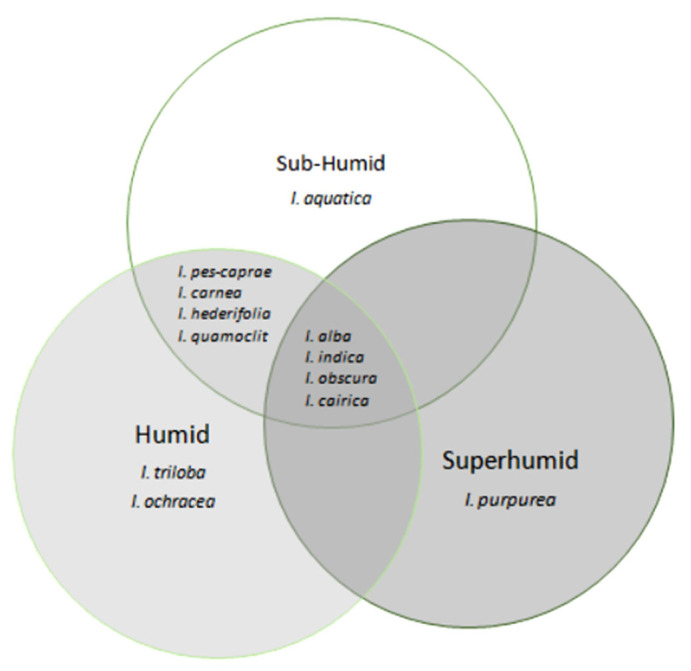
Distribution of Ipomoea species in the different agro-climatic regions of Mauritius.

Figure 1 and Figure 2 show the distribution patterns, and Figure 3, the altitude preferences of 14 *Ipomoea* species and 2 subspecies in Mauritius, respectively. The distribution of most *Ipomoea* species exhibited no correlation with soil type, except for *Ipomoea pes-caprae*, which was encountered on sandy beaches. Figure 4 shows the species classification based on different agro-climatic conditionss, primarily determined by rainfall and moisture levels, and Table 1, the number of sites and percentage occurrence of the respective species. Despite their presence across various climatic zones on the island, 52% of all *Ipomoea* species were observed to dominate humid zones. Overall, the distribution pattern suggests a nuanced relationship between *Ipomoea* species and rainfall levels. While some species predominantly inhabit low rainfall regions, others, like *I. indica*, exhibit adaptability to moderate rainfall environments. The prevalence of *Ipomoea* individuals in humid zones further underscores their affinity toward moisture-rich habitats. The distribution of individual *Ipomoea* species is further discussed under the ecogeographic conspectus (see below).

The highly compressed boxes of *I. purpurea*, *I. quamoclit,* and *I. herderifolia* (Figure 3) are mainly due to their rarity over the island where less than four populations were encountered and occurred at similar altitudes. Similarly, *I. triloba* was uncommon but was seen at different altitudes ~30, 300, and 380 m. *I. ochracea* population was mainly found in the Moka district at a moderate altitude averaging 301.02 (±13.18) m. Occasionally, the species was also seen at higher and lower altitudes.

### 2.4. Relationship between Species Distribution and Geographical Area

**Figure 5:**
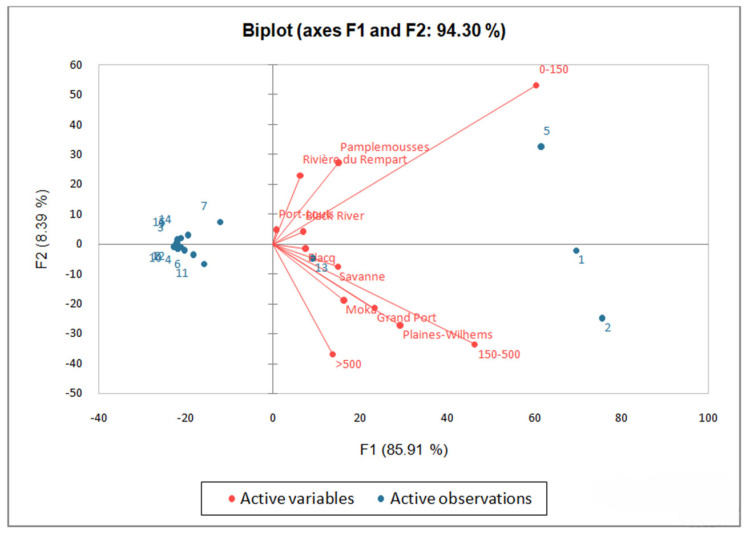
PCA ordination diagram and plots of studied *Ipomoea* in relation to abiotic indexes.

A PCA was conducted to determine the relationship between species distribution and geographical area. The PCA ordination diagram and plots of the studied *Ipomoea* in relation to abiotic indexes (Figure 5) showed significant variation across distribution status. The PCA reveals that a large portion of the dataset’s variance (85.91%) is explained by the first principal component (F1). The first axis mainly represents the gradient from a tropical humid zone to a tropical super-humid zone, and then to the drier sub-humid part of Mauritius. The species were closely linked to geographic regions with different climatic conditions. This might suggest that the species distributions are not random and that climatic and geographic variables present in different districts play a crucial role in shaping species distribution patterns. Figure 5 shows that the PCA plot categorizes geographical areas based on climatic and altitudinal characteristics. For example, Pamplemousses, Rivière du Rempart, Black River, and Port Louis, delineated in the upper right quadrant, are categorized by the lowland and plains. Normally, these regions are known to be higher temperature zones with lower rainfall and moisture indices. Hence, the position of *I. obscura*, denoted by five, signifies that the species, compared to other species, have a higher occurrence along those components compared to other data points, more particularly around Pamplemousses and Rivière du Rempart localities.

Data points, 1 (*I. alba*) and 2 (*I. indica*) located in the lower right quadrant of a PCA plot, represent a distinct subset of the dataset. It suggests that *I. indica* has more individuals occurring at higher altitude ranges. Being situated in a particular quadrant does not exclude the incidences along other components but highlights its prevalence at a particular one. Moreover, it is found further away from the point of origin because, during this study, it was encountered more frequently than the others. *I. cairica* (4) is the fourth most commonly encountered species but at a smaller number of individuals compared to 1, 2, and 5. Moreover, the majority of incidences occurred at moderate altitudes. The variable points categorized by a negative score, on the left side of the PCA plot, mean that they have lower incidences along those components compared to other data points, hence, contribute less to the overall variance in the dataset. This makes it difficult to fit them into any grouping category. The significance of Pillai’s trace and Wilk’s Lambda (*p* < 0.05) suggests that geographical area, which is characterized by its climatic, edaphic, altitude, and temperature, has an effect on species distribution.

### 2.5. Regional Richness

**Figure 6:**
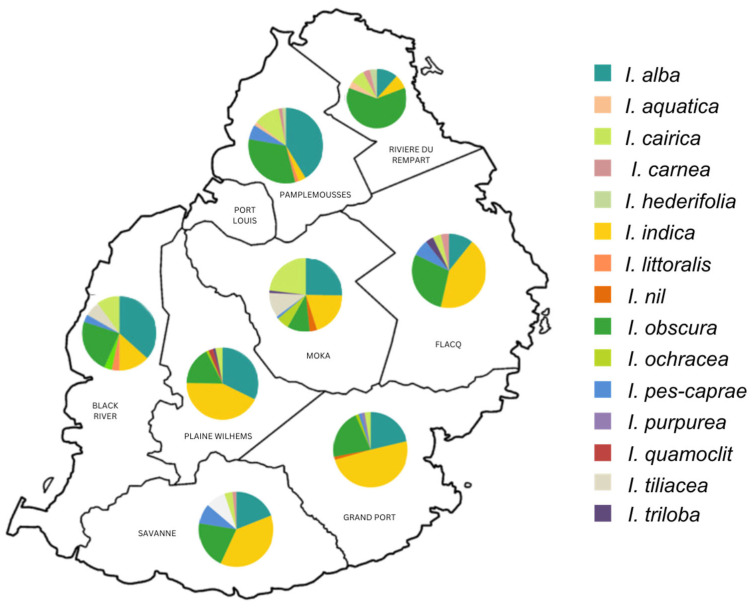
District-wise distribution of the species of *Ipomoea* within Mauritius.

Different species were found in the sampled localities within the nine districts of Mauritius. Based on the species richness values, Table 2 highlights that none of the districts had all of the taxonomic groups. Figure 6 shows that *I. indica* is the most widespread across the southeast regions, including Plaine Wilhems. *I. alba* is the most dominant species in the west and northwest, whereas *I. obscura* is the most dominant in Rivière du Rempart. The least-represented species was *I. purpurea*, which represented only 0.2% of the total individuals identified in this study. Other less frequent species include *I. aquatica*, *I. littoralis*, and *I. quamoclit* with 0.4% occurrence, and *I. hederifolia* and *I. trioba* with 0.6% occurrence.

Table 2 shows Moka and Pamplemousses had the highest beta diversity with nine species occurring within those districts. Additionally, Moka alongside Plaines-Wilhems were among the districts with the most populated localities, which accounted for observations beyond 90 individuals.

The Shannon diversity index indicates that Moka, with the highest index (1.85), has one of the highest alpha diversities in Mauritius. The complementary, 0.84, value of evenness, also validates the region’s high diversity. On the other hand, Black River and Savanne, with the respective, 1.73 and 1.62 indices, also represent significant diversity. However, their equivalent evenness values, 0.83, reveal that the species are distributed in similar proportions in those districts

### 2.6. Ecogeographic Conspectus

**Figure 7:**
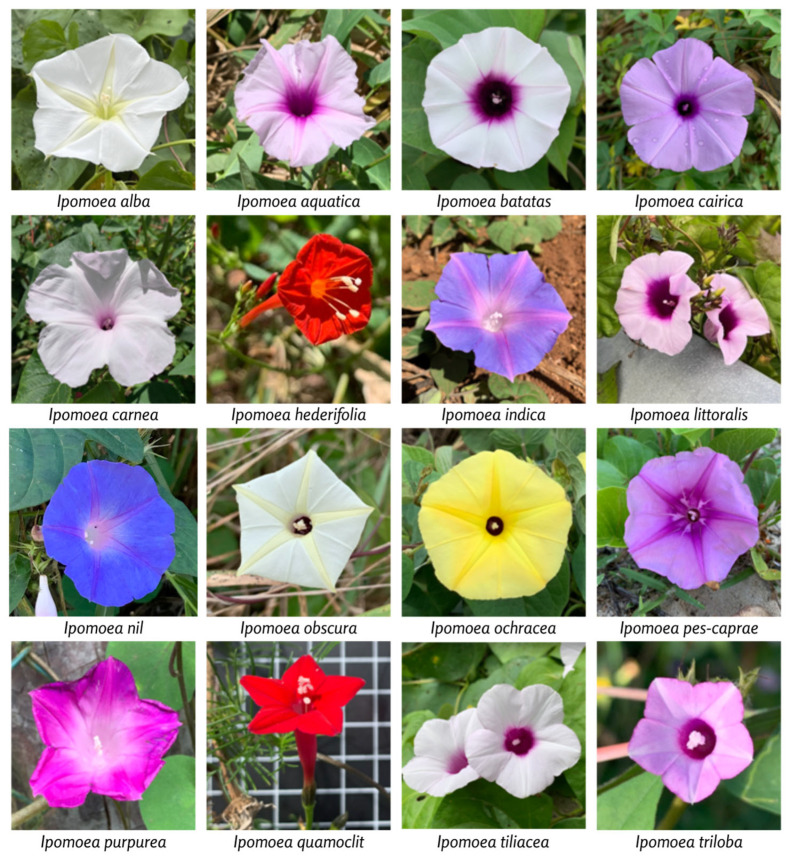
Pictures of *Ipomoea* species encountered during this ecogeographic study.

This conspectus serves as a formal summary of the available taxonomic, geographic, and ecological information pertaining to the target taxon, in this case, the 16 *Ipomoea* species (Figure 7) identified during the fieldwork. It is organized by species and includes details such as distribution, phenology, altitude, and ecology. More profound taxonomic work on each species has been presented by Bosser et al. [32] in the Flore des Mascareignes.

#### 2.6.1. *Ipomoea alba*

Distinguishing characteristics: Herbaceous annual vine, capable of reaching 5–6 m in length. Stems are glabrous, smooth with milky latex. Leaves entire or three-lobed, ovate, broadly ovate to nearly circular blade, 5–15 (−20) cm by 5–10 (−15) cm, acuminate at the tip, deeply cordate at the base with rounded lobes, sometimes angled; inflorescences are cymose, axillary, with one to a few flowers; sepals are unequal, the outer ones elliptical, acuminate, 6–10 mm long, excluding the thread-like tip 5–9 mm long; the inner ones nearly circular, about 12 mm in diameter, with a triangular tip 3–4 mm long. Large, fragrant flowers. Corolla white, funnel-shaped, opening abruptly at dusk, with a slender tube 7–12 cm long and 5–6 mm in diameter; the limb is wheel-shaped, 8–10 cm in diameter. Stamens inserted toward the upper one-third of the corolla tube; anthers oblong, 4–6 mm long, protruding; filaments slender, not widened, and not hairy-papillose at the base. Stigmas protruding. Ovary conical, hairless. Capsule ovoid, apiculate, 20–25 mm in diameter, on a thickened, club-shaped pedicel; calyx persistent, enlarging. Seeds 4, subtriangular, 10–12 × 7–9 mm, hairless, or bearing small pressed, scattered papillose hairs [32,46].

Distribution. Figure 1a and Figure 6 give the distribution of *Ipomoea alba*, in Mauritius. *I. alba* occupies a large geographical range with varying frequency of occurrence and invasiveness. It was less frequently encountered to nearly absent in the southwest and northeast of Mauritius. In addition, from the herbarium records, we note that this species has historically been recorded in Rose Hill, Phoenix, Tombeau Bay, Grand River North West, Curepipe, Mont Roches, and La Ferme.

Phenology. This vine flowers under low light intensity. Flowering starts at dusk and is at its fullest at night. It also blooms during daytime, on cloudy days, and in extremely shady habitats. Flowering was observed throughout the year and abundant fruits were observed in August–November,

Altitude. 1–662 m. In Mauritius, *I. alba* occurs at a broad range of altitudes (Figure 3), where the lowest recorded altitude was at La Gaulette (1.48 m a.s.l.) and the highest at Grand Bassin (662 m a.s.l.). It predominantly occupies regions between 36.52 m and 173.07 m, representing low to slightly moderate altitudes (Figure 3) regions in Grand Port, Flacq, Pamplemousses, and Black River districts.

Ecology. *I. alba* occurs in a diverse range of habitats, including shrublands, forests, as well as dormant volcano craters (Trou aux Cerfs), river cliffs, agricultural, and other ruderal areas demonstrating its ecological plasticity. *I. alba* thrives in regions with very low to low rainfall and moderate temperature and is highly abundant in the humid zones of Plaine Wilhems (43% occurrence). *I. alba* was found to be very invasive in different habitats such as riverine, gullies, roadside lands, and drainage, as well as in agricultural and ruderal areas, where they tend to engulf other vegetation except *Colocasia esculenta* (L.) Schott species and the tall grasses (*Megathyrsus maximus* (Jacq.) B.K. Simon & S.W.L. Jacobs). In drier areas (Le Morne, Albion, and Grand Baie), the species was found to be less invasive, especially during the dry season. In such habitats, the plants tend to have smaller leaves and are less dense and clumped. Such an environment highlights its resilience and its potential to persist, thus potentially harboring unique drought-resistant plants. At present, the population type of the populations is generally classified as natural, indicating that these wild clumps have established themselves through natural processes such as seed dispersal and the growth of stem fragments.

Conservation status: LC [66].

Origin: Alien [32].

#### 2.6.2. *Ipomoea aquatica*

Distinguishing characteristics: Glabrous, annual or perennial herb with thick, fleshy, spongy, and hollow stems, 2–3 m long, creeping on damp or muddy ground, and floating on water. Leaves with variable blade shape, triangular-acuminate, lanceolate or more or less narrow and linear, arrow-shaped, truncated, cordate, or halberd-shaped at the base, (3-) 5–12 (−15) × 3–7 cm, with rounded or pointed basal lobes, sometimes wavy or toothed; margins above the lobes entire or sometimes coarsely toothed; petiole 3–10 (−20) cm long. Inflorescences with 1–2 or a few flowers in a cyme; peduncles 1–8 (−10) cm long, with 2 bracts at the apex; bracts triangular, 1.5–2 mm long; pedicels 1–4 (−5) cm long. Sepals oval or elliptical, obtuse or sub-acute and with a small sharp point at the apex, slightly unequal, 6–8 mm long, with thin and pale margins. Pink flowers, sometimes white. Corolla funnel-shaped, often with a darker center, 3.5 cm long; limb spread out, 3–4 cm in diameter. Stamens and style included; filaments of the stamens inserted near the base of the corolla, unequal, 5–14 mm long, slightly widened and hairy at the base; anthers 3–4 mm long, arrow-shaped. Ovary conical; style 13–16 mm long. Capsule ovoid or globular, 7–9 mm in diameter. Seeds 4 × 4 mm, with dense, velvety, reddish-brown pubescence (not observed in the Mascarene Islands) [32,46].

Distribution. Figure 1e and Figure 6 depict the distribution of the cultivated species *I. aquatica* across the island of Mauritius. During the study, *I. aquatica* was encountered only twice, at Arsenal and Rivière du Rempart. Despite its rarity in the wild, *I. aquatica* is a locally common green leafy vegetable known under the vernacular name of brède souflette. Historically, the species has been observed near a bridge in Black River.

Phenology. Flowering was observed in September.

Altitude. 16–38 m. In Mauritius, *I. aquatica* was seen at low altitudes (Figure 3), where the lowest recorded altitude was recorded at Rivière du Rempart (16.75 m a.s.l.) and highest at Arsenal (37.22 m a.s.l.).

Ecology. *I. aquatica* was found growing in two areas in an aquatic environment at Rivière du Rempart and in a semi-natural habitat (abandoned marshy plot) at Arsenal where the species was trailing among highly competitive species like *I. alba*, *I. obscura*, *Thunbergia alata* Bojer ex Sims, *Cuscuta campestris* Yunck., and *Megathyrsus maximus*. The species thriving in such a clustered habitat indicates its adaptability in dynamic/challenging environments. The number of encounters is too low to represent the *I. aquatica* population’s climatic preference. However, both observations were based in sub-humid zones with moderate temperatures.

Conservation status: LC [66].

Origin: Alien [32].

#### 2.6.3. *Ipomoea batatas*

Distinguishing characteristics: Creeping herb with edible, tuberous roots, red, white, or less commonly yellowish. Stems and branches are striated, highly branched, angular, becoming somewhat cylindrical, rooting at the nodes, hairless or slightly to densely hairy, green or tinged with reddish, with abundant milky latex. Leaves with a broadly ovate blade, entire, slightly cupsidate at the tip, truncated or broadly cordate at the base, or more or less deeply 3–5 (−7)-lobed or with only a few teeth on the margins in the basal part, 4–14 (−20) × 4–16 cm, hairless or slightly to densely hairy; lower surface dotted with very small glands, sometimes difficult to see; petiole 4–15 (−20) cm long, hairless or hairy. Generally, cultivars with palmatilobed leaves have white flowers, entire leaves have violet or pink flowers. Inflorescences axillary, in cymes of (1-) 2–5 flowers; peduncles 3–10 (−18) cm long, hairless or with bristly hairs; pedicels 3–10 mm long, hairless. Sepals elliptical or oblong, nearly equal, 7–10 mm long, acuminate and with a small sharp point, somewhat papery, hairless or hairy or with a few hairs on the margins. Corolla bell-shaped, about 4 cm long, rarely more, pink, violet, or white, with a darker center; as a general rule, cultivars with palmately lobed leaves have white flowers, while those with entire leaves have violet or pink flowers. Stamens and style included. Ovary hairless or with stiff, erect hairs; style hairless, 1.5–2 cm long [32].

Distribution. *I. batatas* are grown as backyard crops across the island. However, it is mainly cultivated in the northern areas (Figure 1e), for commercial purposes. Populations were also found in the wild and occasionally in abandoned lands. Moreover, 62 accessions of *I. batatas*, consisting of the old and newly introduced varieties, are under ex situ conservation by the Agronomy Department at Nouvelle Découverte. Historical observations of the species were made at Réduit, Rose Hill, Flacq, Maison Blanche, Gabriel Islet, and Îlot Brocus

Phenology. Phenological observations were undertaken at the conservation center. Some accessions exhibited prolific flowering, some did so only sparingly, while some did not flower at all. The flowering accessions blossomed from mid-June to early September, with fruiting observed primarily in August. During the flowering period, observations revealed the active pollination of sweetpotato flowers by bees and moths.

Altitude. 9–499 m. The lowest altitudinal observation was at La Gaulette (9.58 m a.s.l.) and highest at Nouvelle Découverte (498.49 m a.s.l.).

Ecology. The center of *I. batatas* production areas experience moderate temperatures and have fertile agricultural lands. Sweetpotato is produced with low or no input. Farmers depend on natural irrigation (precipitation) for their agricultural activities. In semi-natural habitats, *I. batatas* thrived amidst a mix of vegetation including weeds like *Coixlacryma-job* L., *Megathyrsus maximus*, *Piper betle* (L.), and *Mikania micrantha* Kunth. Sweetpotato vines exhibited a climbing behavior, but a remarkably large number of vines streamed out of the dense area to spread onto free spaces.

Conservation status: Data deficient (DD) [66].

Origin: Alien [32].

#### 2.6.4. *Ipomoea cairica*

Distinguishing characteristics: Twining herb, 2–5 (−8) m long, perennial, with tuberous roots. Slender branches, hairless or with small roughness, sometimes hairy at the nodes. Leaves glabrous, palmately divided almost to the petiole, giving the impression of a palmate leaf; blade nearly circular, 2–8 × 2–9 cm; lobes 5–7, narrowly oval to oval or elliptical, blunt to acuminate and tipped with a small sharp point at the apex, narrowed at the base, the middle lobe larger, 4 × 1.6 cm; petiole slender, 2–6 cm long, often with more or less deciduous pseudo-stipules at the base. Inflorescences axillary, hairless, one–few-flowered in a cyme; peduncles 0.5–7 cm long; pedicels 1–2 cm long; bracts small. Sepals hairless, oval to elliptical, with a sub-acute, obtuse, or rounded tip, tipped with a small sharp point, the outer ones 5 mm long, the inner ones 6–7 mm long, slightly warty on the back with more or less translucent spots; margins pale and membranous. Corolla pale mauve with a purple throat or purple with five paler bands (in the Mascarene Islands; elsewhere, it can also be pink or even completely white), funnel-shaped, 5–6.5 cm long; limb spreading, can be 7–8 cm in diameter; lobes well-marked. Stamens and style included; filaments of the stamens unequal, 5–12 mm long, widened and hairy at the base, inserted about 5 mm from the base of the corolla; anthers 4–6 mm long, arrow-shaped. Ovary conical, hairless; style 10–12 mm long. Capsule globular, 10–12 mm in diameter, with four valves. Seeds 3–4, globular, nearly triangular, 5–6 mm long, hairy, and with longer hairs on the angles [32,46].

Distribution. Figure 1d and Figure 6 show the distribution pattern of *Ipomoea cairica* across different agroecological zones. *Ipomoea cairica* with a less common trait (white flower) was located at Rue de l‘Ambassade, Moka.

Phenology. Flowering was observed throughout the year; however, the intensity of flowering varied seasonally and across different agroecological zones. The abundance of flowers was especially pronounced following periods of rainfall (observed in Moka region). In hot and dry areas like Calodyne, Gros Cailloux, Chamarel, Sebastopol, and Surinam, flowering was observed to be relatively low.

Altitude. 5–406 m. In Mauritius, *I. cairica* occurs at a low to moderate range of altitude (Figure 3), where the lowest recorded altitude was at Mahebourg (5.37 m a.s.l.) and highest at St Pierre bypass road (405.07 m a.s.l.).

Ecology. With 70% occurrence in humid areas, especially in Moka and upper Plaine Wilhems, *I. cairica* demonstrated a preference for humid zones with a moderate temperature (22–23 °C). The species was found in wild, cultivated, and ruderal habitats like rocky surfaces, riverine, cane field edges, roadsides, inhabited areas, unoccupied agricultural research land, bushlands, and grassland. In grasslands, the species prospered among short grasses (e.g., *Stenotaphrum dimidiatum* (L.) Brongn.) but deviated from territories densely occupied by *Megathyrsus maximus*. The species typically preferred and invaded open spaces, by trailing over herbs, shrubs, like *Solanum mauritianum* Scop., *Bougainvillea* species, and trees like *Leucaena leucocephala* (Lam.) de Wit, *Litsea monopetala* (Roxb. ex Baker) Pers., *Dypsis lutescens* (H. Wendl.) Beentje & J. Dransf., *Mangifera indica* L., *Bambusa multiplex* (Lour.) Raeusch. ex Schult. & Schult. f. Moreover, species like *I. ochracea*, *I. alba*, *I. tilliacea*, and other species *Solanum nigrum* L., *Cyperus brevifolius* (Rottb.) Hassk., *Coixlacryma-jobi*, *Oplismenus compositus* (L.) P. Beauv. were observed growing alongside. A unique observation of *I. cairica* with white flowers was made in a highly developed area in Moka, where it was colonizing an abandoned land. This rare genotype is worthy of conservation.

Conservation status: LC [66].

Origin: Alien [32].

#### 2.6.5. *Ipomoea carnea*

Distinguishing characteristics: Herbaceous plant, subshrub, or small shrub with white latex, reaching up to 2 m in height. Stems are hollow, hairless, or very finely downy-velvety. Leaves have an oval blade, 5–15 (−20) × 4–8 (−12) cm, pointed or semi-acuminate at the tip, broadly notched to nearly cordate or truncated at the base; margins entire or slightly wavy; tertiary veins more or less perpendicular to the midvein; midvein bearing two small glands at the base on the lower surface; young leaves downy-velvety on both surfaces; adult leaves hairless on the upper surface; petiole 3–8 (−15) cm long, downy. Inflorescences axillary and terminal, in many-flowered cymes; peduncles 3–10 cm long, downy, becoming hairless and somewhat woody and sturdy; pedicels 0.3–1.5 cm long, downy; bracts oval-obtuse, about 2 mm long, downy, falling off early. Sepals nearly equal, the inner ones slightly larger, nearly circular, rounded, 4–5 mm long, downy on the back. Corolla bright pink, often with a purple center, funnel-shaped, with a tube narrowed at the base, 7–9 cm long, with a spread-out limb 8–12 cm in diameter, downy-silky on the outside on the tube and the median bands of the lobes. Stamens and style included; filaments of the stamens very unequal, 1–2.7 cm long, widened and hairy at the base, inserted 5–6 mm from the base of the corolla, anthers oblong, 6–7 mm long. Ovary downy, as well as the base of the style, which is about 2.7 cm long. Capsule ovoid, mucronate, 15–20 mm in diameter, pale brown, downy at the base, with four valves. Seeds four or fewer, black, hairy-silky. Capsules and seeds not observed in the Mascarene Islands [32,46].

Distribution. The distribution of *I. carnea* is shown in Figure 1e and Figure 6. During this study, five populations of *I. carnea* were located at four localities, namely: Tombeau Bay, Souillac, Bel Air, and Petit Raffray. In 1963, the species was encountered at Montebello, Port Louis.

Phenology. Flowering occurs throughout the year.

Altitude. 6–80 m. *I. carnea* has been rather restricted to the lowlands at altitudes between 6.68 and 79.94 m. The maximum altitude recorded was at Bel Air (79.94 m a.s.l.)

Ecology. *I. carnea* exhibits a preference for low to very low rainfall and moderate-to-high temperature. Two populations were found in sub-humid localities (Tombeau Bay and Petit Raffray) and the remaining were in humid zones where they occupied abandoned lands in inhabited areas. The vines of *I. carnea* grew vertically to several meters and sprawled over other vegetation. Its growth strategy looks aggressive but this species easily cohabited with other vegetation like *Ipomoea indica*, *I. batatas*, *Antigonon leptopus* Hook. & Arn., *Ricinus communis* L., *Cuscuta campestris*, *Musa paradisiacal* L., *Carica papaya* L., *Leucaena leucocephala*, and *Dracaena fragrans* (L.) Ker-Gawl. Despite the presence of competing vine species like *I. indica* and *Antigonon leptopus* Hook. & Arn., *I. carnea* and the other species thrived alongside by forming a dense thicket. However, although the species demonstrated prolific flowering, only a few fruits were noticed.

Conservation status: Unknown [66].

Origin: Alien [32].

#### 2.6.6. *Ipomoea herderifolia*

Distinguishing characteristics: Twining or trailing, slender, annual herb. Stems and branches smooth or finely ridged, hairless or slightly hairy. Leaves with an oval, broadly oval, or nearly circular blade, hairless or rarely with very short hairs on the upper surface and at the base of the petiole, 3–16 × (2-) 3–12 cm, narrowed and acuminate at the tip, cordate at the base, entire or sometimes distinctly three-lobed or angled or coarsely toothed; petiole hairless, (1.5-) 3–10 (−13) cm long. Inflorescences few-flowered, axillary, pseudo-dichotomous; the first branching in a forked cyme with one central flower, the two branches forming simple one-sided racemes of about five flowers each; peduncles 3–20 cm long, often hairy; pedicels erect, 4–8 mm long, reaching 15 mm on the fruit; bracts more or less triangular, 0.5–1.5 mm long, mucronate, falling off early. Sepals hairless, nearly rectangular, more or less rounded or truncated at the tip, and bearing a strong ridge below the tip; the outer ones 2–3 mm long and with a ridge 3–4 mm long, the inner ones slightly longer. Corolla uniformly bright red, rarely white, hairless; tube narrow, slightly widened at the top, 2.5–3 cm long; limb spread out, wheel-shaped, 2–2.5 cm in diameter. Stamens with filaments inserted in the lower one-quarter of the corolla, slender, finely papillose at the base; anthers 1.5 mm long, protruding. Ovary conical, with two chambers, each with two ovules; style slender, with protruding stigmas. Capsule globular, hairless, yellowish-brown, 5–7 mm in diameter, with four valves, the partitions persistent, transparent, with thickened outer margins; calyx persistent, not enlarging, spreading, and then more or less reflexed. Seeds 3–4, blackish, more or less triangular, 3.5–4 mm long, with dense, appressed pubescence [32,46].

Distribution. Figure 1a and Figure 6 show the distribution of *I. herderifolia* in Mauritius. As shown, *I. herderifolia* is mainly located in the lowlands in the northern and southern parts of the island at Triolet, Rivière du Rempart, and Chemin Grenier. A vine was also encountered at Réduit, Moka before being quickly outcompeted. Historically, *I. herderifolia* has been encountered in Petit Raffray, Beau Champ, Saint Aubin, Mahebourg, Terre Rouge, and Île aux Aigrettes. Recently, in 2017 and 2018, it was seen at Grand River North West and Grand Malabar Mountain, respectively.

Phenology. Profuse flowering and simultaneous fruiting were observed in September and October. Exceptionally, the vine encountered in Moka bloomed in June, whereby some fruit formation was noticed.

Altitude. 19–40 m. *I. herderifolia* has a narrow range of altitude. Its lower limit was 19.22 m a.s.l. at Rivière du Rempart and extends to Triolet at 39.31 m altitude (Figure 3).

Ecology. *I. herderifolia* habitats ranged from shrublands in Chemin Grenier to disturbed roadside habitats in Triolet, Rivière du Rempart, and Réduit. Triolet and Rivière du Rempart are classified as sub-humid areas while Chemin Grenier and Réduit are humid areas with moderate temperature. In most areas visited, *I. herderifolia* thrives on the canopy of shrubs and trees like *Leucaena leucocephala*, but seems to compete with species like *Passiflora foetida* L., *Cuscuta* vines, *Hibiscus calyphyllus* Cav., *Vigna radiate* (L.) R. Wilczek, and other weedy plants for space on the ground.

Conservation status: Unknown [66].

Origin: Alien [32].

#### 2.6.7. *Ipomoea indica*

Distinguishing characteristics: Large, twining annual herb with stems bearing long, backward-pointing hairs, more or less dense. Lower leaves with a broadly ovate, entire blade, pointed or acuminate at the tip, cordate at the base with rounded lobes; upper leaves with a three-lobed blade, with oval-pointed to pointed lobes, the middle one larger and narrowed at the base; blade 4–10 (−15) × 3.5–8 (−12) cm, more or less hairy or rarely hairless on the upper surface, hairy to densely pale velvety on the lower surface; petiole 3–10 (−12) cm long, with backward-pointing hairs. Inflorescences axillary, in dense, umbel-like cymes, few to many-flowered; peduncles 5–15 (−20) cm long, with backward-pointing hairs; pedicels 2–10 (−15) mm long, hairy; bracts narrow and linear or those at the base sometimes leaf-like, 1–2 cm long. Sepals oval-pointed, 12–20 mm long, nearly equal or the two inner ones narrower, more or less hairy on the outer surface. Corolla azure blue to violet-purple, becoming purplish-red to pink, with pink bands in the median zone of the lobes, funnel-shaped, 5–7 cm long, hairless, the bud may have a few hairs at the apex; limb abruptly spreading to recurved, reaching 7 cm in diameter. Stamens and style included; filaments of the stamens inserted 4–5 mm from the base of the corolla, unequal, 10–18 mm long, with a slightly widened, hairy base; anthers 4–5 mm long. Ovary hairless; style 2–2.5 cm long. Capsule globular, hairless, brownish, 1.2–1.5 cm in diameter, with four valves, surrounded by persistent and slightly enlarged sepals. Seeds four, globular, nearly triangular, about 5 mm long, blackish, covered with very short velvety pubescence [32,46].

Distribution. Figure 1b and Figure 6 display the widespread distribution of *I. indica* across Mauritius, spanning from the central to the southeast regions of the island. The species is concentrated in the Plaine Wilhems district, with rarity observed in the north and absence noted in the west and northeast. Occurrences of *I. indica* were recorded in Rose Hill and Mahebourg in 1947 and 1951, respectively.

Phenology. Flowers all year round, abundantly in summer (August–November). The passage of heavy rain in December and January decreased flower production. The flowers change color to pinkish shade by the afternoon and die.

Altitude. −0.90 to 730 m. In Mauritius, *I. indica* has a broad range of altitude, occurring at −0.90 m a.s.l. at Quatre-cocos to 729.03 m at Chamouny. Moreover, the left-skewed distribution shows a population concentration between 281.59 m and 439.99 m. The extension of the whisker beyond 600 m indicates instances of individuals thriving at higher altitudes (Figure 3).

Ecology. *I. indica* dominates spaces to varied extents across different landscapes ranging from roadside verges to wild green spaces like shrublands, riverbanks, forest edges, and coastal areas to abandoned agricultural lands. The species showed an affinity for wet climate, demonstrated by the clustered populations in the Plaine Wilhems localities (Figure 1b and Figure 6) with humid to super-humid zones, moderate-to-high rainfall, and moderate temperature. It was particularly abundant in Curepipe, whereby it accounted for 70% of the individuals encountered. It was thriving in inhabited areas, by climbing and trailing over structures such as green hedges, fencing, and electric wires, and also covered abandoned plots and garden areas. In the wild, it exists in shrubby lands, forest edges, and roadsides, where it is exposed to abundant sunlight. However, its presence in the north and along southern coastal regions shows its tolerance for warmer and drier conditions. An extensively large population was observed at Bois des Amourettes and Riambel where the vines smothered and outcompeted tall trees, shrubs, and other vegetation by forming a dense mat. The species population was less populated and invasive, and flowering also was sparse on forest edges and riparian zones with less exposure and at the northern sites. Importantly, *I. indica* with pink shade buds and flowers were observed during daytime (between 14 00 and 16 00 UTC + 04:00) in the southeast areas. Fruits of *I. indica* were not common; abundant clusters of fruits were observed at a greenbelt site in Moka.

Conservation status: Unknown [66].

Origin: Alien [32].

#### 2.6.8. *Ipomoea littoralis*

Distinguishing characteristics: Twining or trailing herb, hairless, or more rarely with sparse hairs. Leaves with an oval, nearly triangular, or nearly circular blade, pointed or somewhat blunt at the tip, cordate at the base with rounded or sometimes somewhat acuminate and wavy lobes, 2–5 (−10) × 1.5–4.5 (−7) cm, membranous or sometimes slightly thick; petiole (1-) 2–5 cm long. Inflorescences 1–3-flowered; peduncles 1–3 cm long; pedicels 1.5–2.5 cm long; bracts narrow, 1–2 mm long, falling off early. Sepals oval to broadly oval, with a short ridge, 6–12 mm long, the outer ones slightly smaller and narrower, rather leathery. Corolla purple or purplish-pink, darker in the throat, funnel-shaped, 3–4 cm long; lobes prominently marked; tube narrowed at the base. Stamens and style included; filaments of the stamens unequal, 6–11 mm long, with a widened, hairy base; anthers over 2.7 mm long. Ovary with two chambers, each with two ovules, hairless, not acuminate; style 1.8–1.9 cm long. Capsule globular, hairless, about 1 cm in diameter. Seeds four, hairless, black, 3.5–4 mm long (not observed in the Mascarene Islands) [32,46].

Distribution. Two populations of this *Ipomoea littoralis* were encountered in the south of Mauritius, at Chamouny, and in the north at Pamplemousses (Figure 1e).

Phenology. Flowering was observed in September–October.

Altitude. 78–264 m.

Ecology. *I. littoralis* occurs along roadsides, on fences, and on abandoned lands in the two areas identified above, where they were found to growing in association with *Miconia crenata* (Vahl) Michelang, *Oplismenus compositus*, *Bidens pilosa* L., *Ravenala madagascariensis* Sonn., *nephrolepis cordifolia* (L.) C. Presl, *Psidium guajava* L., *Latana camara* L., and vine species like *Mikania micrantha.*

Conservation status: LC [66].

Origin: Alien [32].

#### 2.6.9. *Ipomoea nil*

Distinguishing characteristics: Annual twining herb, with stems bearing short, yellowish, bristly hairs, which point backward. Leaves with entire or three-lobed blades, lobes more or less deep, acuminate at the tip, base cordate, 2–5 (−12) × 2–5 (−11) cm, hairy on both surfaces; petiole hairy, 2–4 (−9) cm long. Inflorescences with one-to-few flowers in a cyme; peduncles (0.5-) 2–5 (−10) cm long, bristly-hairy; pedicels 0.5–1 cm long, hairy. Sepals narrowly oval, prolonged by a long point, 18–30 mm long, with bristly, yellowish hairs, especially on the lower part; point hairless or with fringed margins. Corolla funnel-shaped, pale blue with a white center, turning pinkish-violet, 5–6 cm long, hairless. Stamens and style included; filaments of the stamens 1.5–2 cm long, hairy at the base; and anthers about 2.5 mm long. Ovary conical, hairless, prolonged at the apex by a beak; style about 2 cm long. Capsule nearly globular, 1 cm in diameter, with a long, persistent beak, generally with three chambers and three valves. Seeds about 5 mm long, blackish, with short velvety pubescence [32,46].

Distribution. *Ipomoea nil* does show a pattern of distribution (Figure 1e). It occurred at varied climatic conditions at Triolet, Vacoas-Phoenix, Réduit, Bois Cheri, and Verdun. In 1976, *I. nil* was observed in Black River.

Phenology. Flowering was observed in August–January.

Altitude. 44–435 m. In Mauritius, *Ipomoea nil* lowest occurrence was at 43.05 m at Triolet and maximum at Bois Cheri.

Ecology. *I. nil* thrived in diverse habitats ranging from urban areas, roadside, woodlands, to riparian areas, where they grow in association with plants such as *Dypsis lutescens*, *Litsea monopetala*, *Panicum maximum* Jacq. bushes, and scattered *Leucaena leucocephala* trees. It forms a canopy on thickets fully exposing itself to sunlight. In riparian habitat, the species trailed among grasses such as *Colocasia esculenta*, *Merremia tuberosa* (L.) Rendle, *Mikania micrantha*, and the nearby concrete surfaces.

Conservation status: Unknown [66].

Origin: Alien [32].

#### 2.6.10. *Ipomoea obscura*

Distinguishing characteristics: Perennial twining herb, 1–2 m long, with slender stems and branches, prostrate or climbing, hairless or slightly hairy. Leaves with a broadly ovate to nearly circular blade, acuminate or with a small sharp point at the tip, with a sharp or blunt point, broadly cordate at the base with rounded lobes, 3–9 × 2.5–8 cm, entire or with slightly wavy margins, hairless or with scattered hairs on both surfaces, sometimes only fringed on the margins; petiole 1.5–5 (−9) cm long, hairy. Inflorescences axillary, 1–2 to few-flowered in a cyme; peduncles generally solitary, 1–5 (−10) cm long, hairless or hairy, with small triangular bracts at the apex, 1–2 mm long; pedicels 1–2 cm long, thickened toward the apex, hairless or hairy. Sepals oval, pointed, or obtuse and mucronate at the tip, nearly equal or the outer ones slightly shorter, 3–5 mm long, hairless or slightly hairy; margins thin and median parts thicker, sometimes warty. Corolla bell-shaped, white or pale yellow, with a yellowish band in the middle of the lobes, sometimes with a dark purple center, 1.5–2.5 cm long; limb spread out, 1.5–2 cm in diameter, with prominently marked lobes. Stamens and style included; filaments of the stamens inserted near the base of the tube, unequal, 3–6 mm long, slightly widened and hairy at the base; anthers 1.7–1.8 mm long, arrow-shaped. Ovary conical, hairless; style 6–8 mm long. Capsule globular, apiculate, 6–10 mm in diameter, pale yellow, hairless; calyx persistent at the base, finally more or less reflexed. Seeds four, more or less hemispherical or shaped like a quarter of an orange, nearly triangular, 3.5–4.5 mm long, covered with very short, dense, greyish velvety pubescence [32,46].

Distribution. Figure 1c and Figure 6 show the ubiquitous distribution of *Ipomoea obscura* in Mauritius. Both illustrations demonstrate high occurrences in the northern part of the island, in Pamplemousses and Rivière du Rempart districts. However, the species skipped the low-temperature zone of the island. The species historically occurred at Beau Champ, Pamplemousses, and Pointe aux Sables.

Phenology. Blooms throughout the year, during the afternoon field work, around 14:00 it was noticed that all flowers were closed, except some populations being shaded by other plants were still wide open.

Altitude. 5–392 m. *I. obscura* occurred at low-to-moderate range of altitude; 50% of the individuals were found between 15 and 43 m altitude. Its lower limit at 4.46 m a.s.l. Mahebourg and extends to 392 m at Vacoas-Phoenix (Figure 3).

Ecology. *I. obscura*, the third most encountered species (Figure 1) predominated in sub-humid regions with low to very low rainfall and warm temperatures. Their occurrence ranged from cultivated and disturbed to wild habitats. It trailed along sugarcane field edges, thickets, and unoccupied residential plots, as well as over fences and other man-made structures. It grows in association with (*Waltheria indica* L., *Mimosa pudica* L., *Senna alata* (L.) Roxb., *Ricinus communis* L., *Euphorbia lacteal* Haw., *Dypsis lutescens,* and Citrus species. *I. obscura* was particularly abundant, showing high invasiveness in northern sites like Petit Raffray, Rivière du Rempart, Goodlands, Cap Malheureux, Grand Baie, Terre Rouge, Roche Noire, and Poudre d’Or. Exceptionally, Grand River South East and Moka sites also harbored large populations. Generally, *I. obscura* established populations without outcompeting other vegetation. It co-existed with vines like *Passiflora foetida*, *I. cairica,* and *I. indica*.

Conservation status: Unknown [66].

Origin: Alien [32].

#### 2.6.11. *Ipomoea ochracea*

Distinguishing characteristics: Twining, trailing, or climbing perennial herb. Stems are hairy. Leaves have an entire, broadly oval blade, pointed or somewhat acuminate at the tip, cordate at the base with rounded lobes, 5–9 (−10.5) × 4–8 (−9) cm; both surfaces with short hairs on the veins or the lower surface is entirely hairy; petiole 1.5–6 (−8) cm long. Inflorescences axillary, one–few-flowered, in a cyme; peduncles 1–5 cm long, hairy; pedicels 0.5–4 cm long, thickened at the apex, hairless. Sepals oval, pointed and mucronate at the tip, 3.5–6 mm long, hairless, with thin margins. Corolla yellow with a purple center, funnel-shaped, 3–4 cm long. Stamens and style included; filaments of the stamens unequal, 4–10 mm long, widened, and with small projections at the base on the edges; anthers about 3.5 mm long. Ovary globular, hairless, prolonged by a beak, gradually passing into the style; style (with the beak) 10–11 mm long, hairless. Capsule conical, 12 mm long and 10 mm in diameter, with four valves, hairless, tipped at the apex by the persistent beak. Seeds nearly triangular, 5 × 4 mm, blackish, hairless but with short pubescence around the hilum [32].

Distribution. Figure 1e and Figure 6 show the distribution of *Ipomoea ochracea* on Mauritius. The 7 populations of *I. ochracea* were restricted to Grand River South East, L’escalier, and Réduit localities.

Phenology. Flowering was observed in July–November. Abundant fruit clusters were observed in the full summer season (particularly in November).

Altitude. 32–305 m. In Mauritius, the species altitudinal range varied from low to moderate (Figure 3), with the lowest occurrence at Grand River South East at 31.67 m a.s.l. and the highest at Réduit at 304.53 m a.s.l.

Ecology. *I. ochracea* showed a preference for specific environmental conditions like humid areas with low rainfall and moderate temperature. It demonstrated varying levels of invasiveness across different habitats such as riparian zones, roadside areas, and neglected agricultural research territories, where they grew in association with various species such as *Megathyrsus maximus*, *Calophyllum species*, *Cardiospermum halicacabum*, *Schinus terebinthifolius*, *Brachiaria mutica*, *and I. triloba*, *Mikania micrantha*, *I. cairica*, and *I. alba* vines.

Conservation status: LC [66].

Origin: Alien [32].

#### 2.6.12. *Ipomoea pes-caprae*

Distinguishing characteristics: Perennial, hairless herb, trailing for several meters to 10–20 m or more, sometimes rooting at the nodes and forming thick mats. Leaves held vertically, with a generally thick blade, broadly oval, obovate, nearly circular or nearly square, (3-) 5–8 × (3-) 4–7 cm, wedge-shaped to truncated at the base, notched or two-lobed, more rarely entire, at the apex; lobes rounded, 5–10 mm long; network of veins with nearly rhombic meshes; petiole 1–6 (−10) cm long. Inflorescences axillary, one–few-flowered in a cyme; peduncles erect, (1-) 3–10 (−15) cm long; pedicels 1–3 (−4.5) cm long. Sepals oval or elliptical, blunt and with a small sharp point at the tip, 5–10 (−12) mm long, the outer ones slightly smaller. Corolla mauve, with a darker center, funnel-shaped, 3.5–5 cm long; limb spread out, 4 cm in diameter, with prominently marked, triangular, blunt lobes. Stamens and style included; filaments of the stamens unequal, 5–10 mm long, widened at the base and hairy on their lower half; anthers 3–4 mm long. Ovary hairless; style 12–13 mm long. Capsule globular, 12–18 mm in diameter, leathery, tipped by the persistent remains of the style, surrounded by the persistent, slightly enlarging calyx. Seeds about a quarter of a sphere, about 7 mm long, dark brown, with fairly long pubescence, especially on the angles [32,46].

Distribution. Figure 1e and Figure 6 give the distribution of *Ipomoea pes-caprae* on Mauritius. The species predominates coastal areas, mainly sandy beaches. However, it also spreads to land areas near the coast and was further uncommonly observed in weedy habitats at Moka and Chemin Grenier. Herbarium historical records show the occurrence of the species in Britannia, Poste Lafayette, Grand Gaube, Pointe D’Esny, Grand Sable, La Cambuse, Bain Boeuf, Quoin Bluff, Round Island, Gabriel Islet, Flat Island, and Îlot Marianne.

Phenology. Flowering was observed in May-December. The afternoon study revealed that past noon the flowers started closing and by 15 30 they were closed.

Altitude. 2–312 m. *I. pes-caprae* is essentially a lowland species and the coastal occurrences were restricted to the range of 2.47 m at Tombeau Bay to 22.87 m at Pomponette Beach. Populations were uncommonly found at Chemin Grenier (31.97 m a.s.l.) and State House Ave, Moka (311.31 m a.s.l.) (Figure 3).

Ecology. *I. pes-caprae* occurred at moderate to high temperatures and at very low to low rainfall. At Trou D’Eau Douce and Riambel sites, most vines have been observed to actually grow toward the sea. Populations on beaches have been observed growing among the vegetation, namely, *Cynodon dactylon* (L.) Pers., *Stenotaphrum dimidiatum*, *Hydrocotyle bonariensis* Comm. ex Lam., *Canavalia rosea* (Sw.) DC., and *Scaevola taccada* (Gaertner) Roxb. Sometimes the populations appeared to co-exist (Tamarin beach), but sometimes, when *I. pes-caprae* invades areas forming a dense mat like at Quatre Cocos beach, less space is left for other vegetation and they are eventually outcompeted by the latter. Otherwise, *I. pes-caprae* occurrences have also been observed at tide line level, on gravels contouring the coastline, and farther on cliffs. Inland habitats alongside roads close to the sea are common, but occurrence in inland areas far from the sea, like in Moka, is rare. The population at Moka includes only a few dispersed plants growing erect without demonstrating invasive tendencies.

Conservation status: LC [66].

Origin: Indigenous [32].

#### 2.6.13. *Ipomoea purpurea*

Distinguishing characteristics: Annual, twining herb. Stems cylindrical, with short hairs and longer bristle-like hairs, more or less pointing backward. Leaves with a broadly oval to nearly circular blade, entire or more rarely three-lobed, acuminate at the tip, cordate at the base with rounded lobes, 4–15 × 2.5–14 cm, with short, more or less appressed, scattered hairs on the upper surface, more concentrated on the veins on the lower surface; petiole 2–15 cm long, with the same type of hair as the stems. Inflorescences axillary, one–few-flowered in a cyme; peduncles 2–5 (−18) cm long, with backward-pointing hairs; bracts linear, 4–6 mm long, bristle-tipped; pedicels 8–15 mm long, curved in the bud, reaching 20–25 mm long in fruit. Sepals narrowly oval to elliptical or oblong, 10–15 mm long, slightly increasing in size, nearly equal but the inner two narrower, hairy, and with long bristles, more or less dense on the lower half and along the margins. Corolla funnel-shaped, hairless, pink or violet, often paler on the outside and with darker bands in the middle part of the lobes, more rarely white or white with five pink spots in the throat, 4–5 cm long. Stamens and style included; filaments of the stamens unequal, slightly widened, and long-hairy at the base. Ovary hairless, with three chambers; style 2–2.5 cm long. Capsule globular, 10–12 mm in diameter, hairless, yellowish, with three valves, the three partitions transparent, persistent, with thickened edges. Seeds six or fewer, shaped like a grape seed, 3–5 mm long, black, appearing hairless but with a finely papillose surface, especially toward the hilum [32,46].

Distribution. Figure 1e and Figure 6 show a single population of *Ipomoea purpurea* was encountered in the district of Grand Port at Union Park. In 1963, a population of the species was recorded at Barkly, according to herbarium records.

Phenology. Flowering was observed in September.

Altitude. 387 m a.s.l.

Ecology. Union Park lies in the super-humid zone with high rainfall and annual mean temperature within 20–21 °C range. *I. purpurea* was thriving in a habitat, characterized by a mix of ruderal vegetation, including species like *Mikania micrantha*, *Litsea monopetala*, *Pandanus*, *Liriope*, *Ophiopogon*, *Heliconia,* and *Mangifera indica*. In this ruderal habitat, *I. purpurea* particularly competed with another vine *Mikania micrantha* for space, but co-existed together, suggesting a degree of ecological tolerance.

Conservation status: Unknown [66].

Origin: Alien [32].

#### 2.6.14. *Ipomoea quamoclit*

Distinguishing characteristics: Twining herb with slender stems. Leaves with a pinnately divided blade, hairless, 3–5 (or more) × 2–3 (−5) cm, divided nearly to the midrib into 10–15 (−18) pairs of linear lobes, 10–20 × 1–2.2 mm, nearly opposite, the lower ones may be forked at the tip, the upper ones gradually shorter, with a rounded tip, the terminal lobe mucronate; petiole 1–2 (−3) cm long, with a pair of divided pseudo-stipules at the base, similar to the leaf blade, 5–12 mm long, more or less stalkless, falling off early. Inflorescences axillary, cymose, one–few-flowered, hairless; peduncles slender, 2–10 cm long, exceeding the leaves, with two triangular bracts at the apex about 1 mm long; pedicels 1–2 cm long, slender, widened at the apex. Sepals oval, mucronate, with a small sharp point below the tip, nearly equal or the inner ones slightly larger, 4–6 × 2.5–3 mm, not increasing in size, hairless, with transparent margins. Corolla salver-shaped, bright red, 3–3.5 cm long; tube nearly cylindrical, slightly widened at the apex, 2–4 mm in diameter; limb abruptly spreading and more or less rotate, 1.5–2 cm in diameter, with five more or less pointed lobes, 5 mm long. Anthers and stigmas included. Stamens inserted 5–6 mm from the base of the corolla; filaments hairy at the base; anthers about 1 mm long, arrow-shaped. Ovary hairless, with four chambers, each with one ovule, gradually narrowed at the apex. Capsule ovoid, 6–9 mm long, with four valves. Seeds four, black, ovoid to ellipsoid, 5–6 mm long, bearing tufts of tiny papillose hairs [32,46].

Distribution. Figure 1e and Figure 6 depict the occurrence of *Ipomoea quamoclit* on Mauritius. During the study, encounters with this species were relatively rare, observed only twice. The populations were situated in Vacoas-Phoenix within roughly 1 km distance. In 1964, the species was observed in Phoenix, according to herbarium records.

Phenology. Flowers were observed in August.

Altitude. 383–388 m a.s.l. The highly compressed box of *I. quamoclit* in Figure 3 is mainly due to its rarity over the island where the encountered individuals number remains at only two, and they occurred at the same range of altitudes.

Ecology. Vacoas-Phoenix is a humid area with low rainfall and experiences moderate temperatures. The populations were found on the edge of cultivated fields and roadside. On the farm edge, the species did not show aggressive growth and invasive tendencies. The vines, full of fruits and seeds, used plants like *Bidens pilosa* and *Phyllanthus niruri* as support.

Conservation status: Unknown [66].

Origin: Alien [32].

#### 2.6.15. *Ipomoea tiliacea*

Distinguishing characteristics: Twining herb, slender. Stems hairless to somewhat hairy, reaching several meters in length. Leaves with entire blade, hairless or sometimes with appressed hairs, broadly ovate to nearly circular, 5–15 × 3–10 cm, cordate at the base, somewhat narrowed and acuminate at the apex, the apex generally blunt and with a small sharp point; petiole slender, 3–10 (−14) cm long, sometimes slightly hairy toward the apex. Inflorescences axillary, few-to-many-flowered, cymose; peduncles 4–15 cm long, hairless; pedicels 5–12 mm long, hairless; bracts narrowly triangular, small, deciduous. Sepals oval or elliptical, mucronate, somewhat membranous, hairless, or fringed on the margins, 5–10 mm long. Corolla pink or purple, with a darker center, funnel-shaped, 4–5 cm long, hairless. Stamens and style included; filaments of the stamens unequal, 7–16 mm long, with a widened, hairy base; anthers about 4.5 mm long, arrow-shaped. Ovary hairless; style 22–23 mm long. Capsule globular, with four valves. Seeds four, hairless or hairy on the angles. Capsule and seeds not observed in the Mascarene Islands [32,46].

Distribution. Figure 1e and Figure 6 give the distribution of *I. tilliacea* on Mauritius. In Mauritius, the species has been encountered mainly at Réduit and subsequently at Bagatelle, St Pierre bypass road, and Plaine Champagne road. Historically the species was found at Sorèze Falls and Pamplemousses Botanic Garden.

Phenology. Flowers were observed in August and September.

Altitude. 254–547 m. Most occurred within the range 253.27–312.55 m a.s.l. However, populations were also seen up to 405.71 m. Displayed as an outlier, the species was also encountered at 546.92 m at Plaine Champagne Road (Figure 3).

Ecology. *I. tilliacea* populations thrived in various habitats, including riparian areas, thickets, and ruderal areas. They demonstrated invasive potential in humid regions with low rainfall and moderate temperatures, covering the trees’ canopy and grasses. In a ruderal part in the vicinity of a river near human settlement in Réduit, the species completely overtook the vegetation and it grew with *I. cairica* and not far was an *I. alba* population. In a roadside thicket, the population thrived among numerous weedy plants like *Verbena officinalis* L., *Clibadium surinamense* L., *Sinapis arvensis* L., *Megathyrsus maximus*, *I. cairica*, *conyza species,* and *Stenotaphrum dimidiatum* (L.) Brongn. as understory. The species extended its vines deep inside the thicket amongst other types of shrubs and tree vegetation.

Conservation status: LC [66].

Origin: Alien [32].

#### 2.6.16. *Ipomoea triloba*

Distinguishing characteristics: Annual twining herb, climbing, sometimes trailing. Stems 1–3 m long, hairless or with scattered bristly hairs. Leaves with a broadly oval to nearly circular blade, 2–8 × 2–7 cm, entire to deeply three-lobed, lobes rounded or pointed, the basal ones sometimes angled with lobules, the middle one cupsidate; base broadly notched to cordate; both surfaces hairless or with scattered hairs; petiole (1.5-) 3–10 cm long, slender, hairless or with scattered hairs. Inflorescences axillary, one–few-flowered, more or less umbel-like; peduncles 1–10 cm long, hairless or with a few bristly hairs; pedicels 2.5–8 mm long, warty. Sepals slightly unequal, oval, elliptical to oblong, mucronate, 5–8 mm long, the outer three slightly smaller, hairless or slightly bristly on the back, with fringed margins; the inner two slightly wider, hairless or loosely hairy, with smooth margins. Corolla pink or lavender, with a darker center, funnel-shaped, about 2 cm long; limb with short, blunt, mucronate lobes. Stamens and style included; filaments of the stamens 6–7.5 mm long, slightly hairy at the base, inserted about 4 mm from the base of the corolla, and anthers about 1 mm long. Ovary long-hairy; style hairless, about 1 cm long. Capsule globular, tipped at the apex by the remains of the style, surrounded by the persistent calyx, 5–6 mm in diameter, bearing bristly hairs at the apex with a swollen base. Seeds are nearly triangular, about 3.5 mm long, black, and hairless [32,46].

Distribution. Figure 1e shows the occurrence of *Ipomoea triloba*, at three sites, namely, Réduit, Vacoas-Phoenix, and Grand River South East, on Mauritius. One population occurred on the roadside and two were found on the edge of cultivated areas. Historically, the species was found at Belle Vue, Beau Champ, Saint Antoine, Pailles, and Port Louis.

Phenology. Flowers and fruits were observed in June–August.

Altitude. 32–382 m. In Mauritius, *I. triloba* is distributed in low-to-moderate altitudes, occurring at 31.67 m at Grand River South East to 381.89 m at Vacoas-Phoenix (Figure 3).

Ecology. *I. triloba* was pictured as a rare species, which strived along roadsides (Vacoas-Phoenix) and on agricultural fields climbing on electric poles (Réduit) and young sugarcane crops (in Grand River South East). This non-invasive species with abundant fruits was easily outcompeted by the rapid growth of *Cynodon dactylon* and was also prone to occasional chemical treatments and weeding.

Conservation status: LC [66].

Origin: Alien [32].

## 3. Materials and Methods

### 3.1. Study Area

The island of Mauritius has been set as the geographical breadth for this study. Mauritius is a small island covering 1865 square km, located in the southwestern part of the Indian Ocean, approximately 900 km east of Madagascar.

### 3.2. Ecogeography Study Methodology

A slightly modified version of the ecogeographic study methodology as described in Maxted et al. [67,68] was used in this study. It consists of the selection of target taxon, identification of taxon expertise, identification of taxon collections, listing of germplasm conserved, media survey of geographical, ecological and taxonomic data, delimitation of the target area for study, collection of ecogeographic data, analysis of geographic and ecological data, ecogeographic conspectus, and identification of conservation priorities. However, the study did not include project commissioning and use of a Database Management Systems (DBMS) package as suggested by Maxted [67] (see the ecogeographic database Section below).

#### 3.2.1. Identification of Taxon

During the initial phase, a literature review was conducted to identify sources of information on the occurrence of *Ipomoea* species in Mauritius [13,32,51,69,70]. Gene bank collections and The National Herbarium of Mauritius were also consulted. Further information on the targeted species was obtained from reliable online sources like the Global Biodiversity Information Facility (GBIF), Plants of the World Online (POWO), Center for Agriculture and Biosciences International (CABI), Jstor, and SEInet. Living plant images and information from herbaria specimens and field identification characteristics such as habitat preferences available through these sources were collected and proved to be valuable for the ecogeographic study. Through these inventories, a list of these *Ipomoea* species with their respective description and identification leaves, flowers, and fruit images was made. Bosser et al. [32] taxonomic work served alongside other relevant literature as references to differentiate between species.

In addition to the above, local experts from key institutions including Mauritius Herbarium, Department of Agronomy (which houses the National Gene Bank), Food and Agricultural Research and Extension Institute (FAREI), University of Mauritius, National Parks and Conservation Service, Forestry Service, and others were consulted to gather additional information on the *Ipomoea* species from Mauritius.

#### 3.2.2. Fieldwork

**Figure 8:**
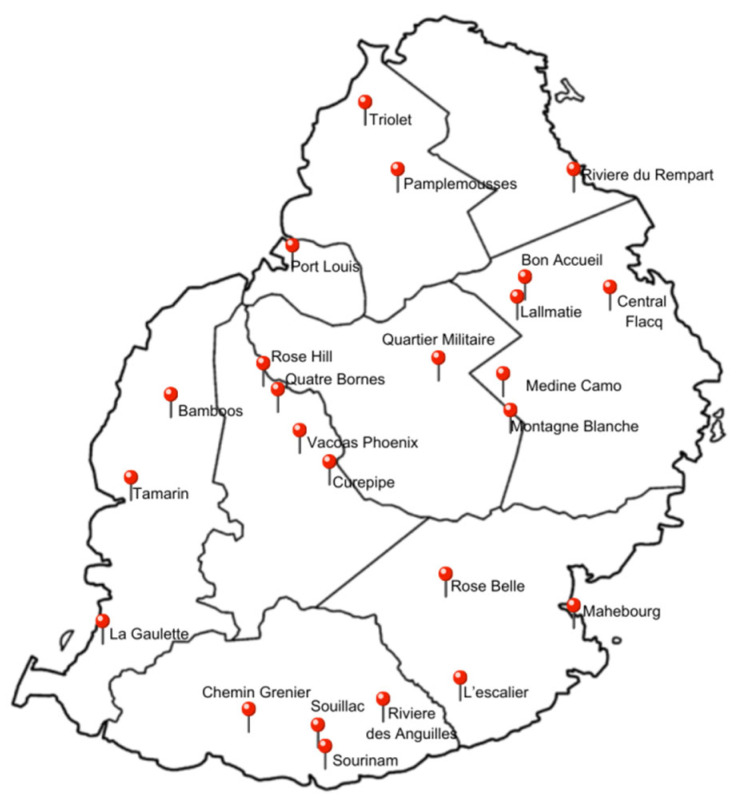
Wet markets visited in 2022.

The study relied mainly on ground-truth data obtained through field explorations. An island-wide survey was conducted in more than 24 wet markets (Figure 8) and other retail points on the survey routes, during the festival of lights (Diwali) season, in October 2022, when there was an increased production of sweetpotato on the island. Information on local varieties of cultivated *Ipomoea* species (*I. batatas* and *I. aquatic*) was gathered during these field visits from actors along the value chain, including auctioneers, wholesalers, and retailers. This survey helped trace back farmers. Data collected from farmers were used to locate the main areas of cultivation of the commercial species.

Fieldwork was carried out across all districts (the formal categorization of land in Mauritius) including Pamplemousses, Riviere du Rempart, Flacq, Grand Port, Savanne, Black River, Plaine Wilhems, Moka, and Port Louis. Climatic differences may exist within the districts due to variations in elevation, proximity to the coast, and other factors. Google Earth Pro [71] was used to develop reference maps covering the island’s distinctive features, including topography, altitude, and climatic variations. More than 90 areas (Table 3) were delimitated, ensuring that all agro-climatic regions throughout Mauritius were prospected for the putative presence of *Ipomoea* species. In November 2022 and August–October 2023, the species were sampled from more than 450 sites. These sites represent different habitats such as inhabited localities, agricultural land, abandoned lands, green space, and river reserves, which are predicted types of habitats usually preferred and inhabited by *Ipomoea* species. In green spaces and river reserves, particularly, random walks were undertaken to spot the species.

Identification of the encountered species was mainly through flowers, leaves, and fruits guided by the identification sheet prepared and common reference to online live plant images. Observations were made by maintaining around 1 km distance between species of the same kind. Once individual(s) were encountered, observations were made and recorded on a field sheet, formulated to record various variables in relation to the plants’ ecology, geography, and climatology. Each species sample studied was photographed in its habitat and tagged with an identification number for consultation in times of need.

#### 3.2.3. The Ecogeographic Database

The field study generated a database on the ecology of *Ipomoea* populations. A database was built on IBM SPSS Statistics 21 [72]. This central file contained data gathered based on only ground-truth observations and excluded records obtained from the herbaria and other sources. The *Ipomoea* species habitats were characterized using the classification system and terminologies adopted by Mistura et al. [73] and Alercia et al. [74]. The latitude, longitude, and elevation of the study points were read using a GPS. Thus, data collection at each observation site included species name, geographical location (coordinates, village, region, and district), altitude (ma.s.l.), population type of the population, population distribution pattern, status of the occurrence site, topographic description, and land use.

The study sites were further categorized considering soil groups and under different climatic regimes based on moisture efficiency, rainfall, and temperature. A Soil Map of Mauritius was obtained from Fischer et al. [75] Climate data were retrieved from Dhurmea et al. [76], Halais and Davy [77], and Building Resilience in Indian Ocean (BRIO), climate website [78]. Specifically, a mean annual temperature (1881–2014) map was obtained from BRIO [78] and a spatial distribution of long-term mean rainfall (1981–2010) from a recent publication of Dhurmea et al. [76]. Data on moisture such as Thornthwaite’s Moisture Efficiency Index and Thornthwaite’s Seasonal Variations of Moisture Efficiency were obtained from published maps [77].

Using recorded coordinates, species distribution maps were generated using Google Earth Pro [71]. These maps were then overlaid with moisture, annual rainfall, temperature, and seasonal rainfall maps. Each climatic parameter scale was grouped into the categories: altitude (0–150 low, 150–500 moderate, >500 high), moisture (super-humid >100, humid 20–100, sub-humid −20 to 20), rainfall (600–1400 very low, 1400–2000 low, 2000–3000 moderate, >3000 high), and temperature (high >25, moderate 20–25, low <20). As such, each of the individuals studied was categorized with respect to the distinctive climatic parameters by reading from the overlaid maps. All data were recorded in IBM SPSS Statistics 21 [72].

### 3.3. Statistical Analysis

Variations present between and within the species were analyzed through the evaluation of the multidisciplinary datasets using IBM SPSS Statistics 21 [72], XLSTAT [79], and Microsoft Office Excel [80]. A comprehensive analysis of species distribution patterns across different districts in Mauritius, with a focus on how these distributions are influenced by climatic and topographic factors, was performed. The use of Pillai’s trace, Wilk’s Lambda, Hotelling’s Trace, and Roy’s largest root as statistic tests in MANOVA indicated a thorough examination of the relationship between species distribution and various regional characteristics. To uncover relationships between environmental variables, multivariate statistics were employed. Two principal component analyses (PCA) were conducted using bioclimatic and geographic variables. Regional richness, diversity, and evenness indices were calculated using an Omni calculator [81].

## 4. Discussion

The primary aim of any ecogeographic study is to comprehensively assist in the formulation of in situ and ex situ conservation priorities and strategies [27,67] to help maximize genetic diversity [15]. The present study of the *Ipomoea* species on Mauritius Island has provided useful information on their ecogeographic distribution, as well as on ecology, geography, and taxonomy, to allow conservation priorities to be determined. There have been no previous ecogeographic studies or surveys carried out to determine the distribution of the *Ipomoea* species in Mauritius.

Maxted et al. [68] stressed the importance of gathering information from as many herbaria as possible, as they are essential sources of ecological and geographical information. However, the analysis of the herbarium data on *Ipomoea* species at the Mauritius National Herbarium highlights certain limitations and gaps regarding ecological and geographical information, including soil type, latitude, longitude, habitat type, and altitude. Soil type was absent from the records, while only 12.5% and 37.5% of latitude and longitude and habitat type were recorded, respectively. Moreover, the results also showed that some of the curatorial data, in a few cases, collector’s numbers, species locality, year of detection, and species name, were missing. These hindered mapping and spatial analysis of species distribution and made it challenging to understand and draw conclusions on the ecological preferences and requirements and evaluate the status of the species represented in the herbarium. This information had to be supplemented through extensive field studies based on the localities recorded.

The 14 species and 2 subspecies encountered during the fieldwork represent 37% of the total number of Convolvulaceae species and 70% of the *Ipomoea* species on the island of Mauritius. According to POWO and Bosser et al. [32], the occurrence of *I. venosa*, *I. rubens,* and *I. fimbriosepala* in Mauritius is considered doubtful. The herbarium data revealed that the last record of *I. horsfalliae Hook*. and *I. mauritiana* date back to 1963 and 2009, respectively. *I. horsfalliae*, previously used as an ornamental plant in Mauritius, may have been owned by plant collectors. Bosser et al. [32] alluded to the fact that this plant does not fruit outside its native regions of Jamaica and Puerto Rico. Similarly, Wood and Scotland [82] reported that cultivated *I. horsfalliae* rarely produce fruits. This lack of fruiting might have hindered the natural succession of the species, which led to its disappearance from Mauritius. *I. mauritiana* has been reported to occur in a few river reserves [32]. However, despite our numerous visits to riparian zones, the species was not found. The last herbarium record of this species dates back to 2009 when it was observed at the Bamboo mountain range in the east of the island. Additionally, the herbarium data also show that *I. violaceae* does not occur on the mainland of Mauritius but occurs on islets around Mauritius, which were not visited in the course of this study. Bosser et al. [32] reported that *I. ochracea* (probably of African origin) occurs only in Reunion Island and no specimen from Mauritius was found in the Mauritius National Herbarium. According to POWO, *I. ochracea* occurrence in Mauritius was reported in the early 1800s and lastly in the mid-1900s. During this study the species was identified from Grand River South East, L’Escalier, and Réduit localities (Figure 1e and Figure 6), constituting a first record for Mauritius.

### 4.1. Distribution Pattern

Several factors influence the spatial distribution and behavior of the *Ipomoea* species in Mauritius. Figure 2 indicates that the majority of *Ipomoea* species are adapted to a range of soil conditions. However, they are rare or even absent in leptosols, humic cambisols, ferralic cambisols, chromic cambisols, humic ferralsols, and calcic vertisols. Humic ferralsols are typically characteristic of Mauritius’ forest, characterized by dense, shaded environments and intense competition among plant species. *Ipomoea alba*, *I. indica*, and *I. obscura* exceptionally demonstrate a higher degree of adaptability compared to the other species. The high prevalence of *I. alba* and *I. indica* within settlement areas, illustrated in Figure 2a,b, highlights their adaptability to anthropogenic environments. In contrast, *I. pes-caprae* stands out as a clear exception for its strong tolerance and affinity for sandy beach environments. While Figure 2 provides initial insights into species–soil relationships, a more comprehensive understanding requires consideration of specific soil characteristics (e.g., pH, nutrient content, texture) to elucidate species-specific requirements and determine the precise edaphic factors influencing the observed distribution patterns.

Figure 1, Figure 4 and Figure 5 demonstrate the influence of climatic factors such as temperature and moisture on the species distribution. Figure 4 shows that some species (*I. triloba*, *I. ochracea*) were found in only specific agro-climates, which could have been due to their rarity and recent introduction. Some species were present in two climate categories, while some like *I. alba*, *I. indica*, *I. obscura,* and *I. cairica* were present across all agro-climatic zones. However, the species’ behavior varied based on climate and habitat. *I. alba*, for example, appears to favor habitats that are permanently or seasonally moist [29]. The invasiveness and aggressivity of this species, especially in or near riverine habitats, gullies, and cliffs might pose a problem to the conservation of biodiversity, as they form canopies over trees and minimize sunlight penetration to host plants [83]. Their control will be necessary for the conservation of other riverine and aquatic plant species. Similarly, *I. cairica* showed a preference for humid localities (72% occurrence) where an abundance of flowers was observed to be high following rainfall periods [52]. Some regions experienced more prolific flowering than others. In hot and dry areas like Calodyne, Gros Cailloux, Chamarel, Sebastopol, and Surinam, flowering was observed to be relatively low, resulting in less fruit and seed production, and were less abundant. In contrast, in the humid localities of Moka and Plaine Wilhems, populations were more frequently encountered. The observed variation might suggest a correlation between environmental factors such as rainfall and the reproductive behavior of the species. Apart from *I. alba* and *I. indica*, other *Ipomoea* species were barely encountered in the super-humid zone at less than 22 °C. *Ipomoea* species were rare in Black River, which experiences lower rainfall (600 mm on the coast) due to elevation and position relative to the prevailing winds [75,84,85,86]. According to Wood et al. [29], *Ipomoea* is unable to thrive in arid and cold moist regions.

Overall, the distribution pattern suggests a nuanced relationship between *Ipomoea* species and rainfall, moisture, and temperature levels. These climatic factors are known to influence the growth and spatial distribution patterns of the species [87,88,89,90,91,92]. The prevalence of *Ipomoea* individuals in humid zones particularly underscores their affinity toward moisture-rich habitats. *I. alba*, *I. indica*, *I. obscura,* and *I. cairica* are among those that have an extensive habitat distribution suggesting that they may have a lot of diversity and may contain adapted genes to different agro-climatic conditions and can be useful in breeding [93,94].

Geography was also observed to influence the distribution of species [92,95,96,97,98,99]. The significance of Pillai’s trace and Wilk’s Lambda (*p* < 0.05) suggests that geographical area, which is characterized by its climatic, edaphic, altitude, and temperature has an effect on species distribution. For example, in Mauritius, *I. aquatica* cultures are found exceptionally in plains with standing water [100] where farmers normally cultivate species like *Nasturtium officinale*. *I. carnea* and *I. hederifolia* have been exceptionally found in lowlands. Figure 1 shows the occurrence of abundant populations of *Ipomoea* species in Moka and Plaine Wilhems localities, which could be due to the availability of greenbelts, river reserves, unoccupied lands, agricultural lands, and cleared lands driven by infrastructural development in those regions. In the northern part of the island, fire in the sugarcane fields and weed control might not promote the establishment of *Ipomoea* species. *I. pes-caprae* is frequently encountered on beaches along the coastal region (Figure 3). This species is often used in beach restoration programs, as it is known to minimize dune erosion [101,102,103]. *I. pes-caprae* was also found well above sea level (Figure 3) in inland habitats [101,103], which could be either through a purposeful introduction, accidentally by dispersal, or by spreading due to climate change [103].

Species distribution is frequently influenced by human activities [92,104]. Among the 23 targeted species, only two species, *I. pes-caprae* and *I. violaceae,* are believed to be native to the Mascarene islands, and the remaining species were introduced by humans [32]. Their dispersal is often associated with their use in gardens or in cultivation from which they escaped and became naturalized [46]. Similarly, *Ipomoea* species were observed in a range of habitats such as secondary scrub and cultivation areas to disturbed places near settlements [29]. Some species like *I. aquatica* particularly inhabit fresh water and marshy habitats. However, the *I. littoralis* and *I. tiliacea*, both cited as a typical beach species [29], were actually found on the verge of the road, alongside rivers, riparian zones, and also inland.

The rarity of some *Ipomoea* species (*I. herderifolia*, *I. triloba*, *I. quamoclit*) could be explained by their uncommon use, high competition, and weed control activities in the habitat where they are present. Despite prolific seed production at various sites, populations of *I. herderifolia* and *I. quamoclit* are not widespread. The presence of a large number of dry vines from previous generations indicates that they are regenerating at specific sites, but their rarity on a regional basis might suggest that there is an issue with the seed dispersal mechanism. *I. purpurea* is also found to be rare, but this may be due to taxonomic challenges in distinguishing it from *I. indica* and *I. nil*. According to Shrestha and Rajbhandary [105], these species could be distinguished by their sepals. However, the sepals could show morphological plasticity as noted by Austin [106] and other authors.

### 4.2. Management and Control

The National Invasive Alien Species Strategy for Mauritius [107] highlights the historical introduction of problematic plants during French rule (1721–1810). These introduced species, termed Invasive Alien Species (IAS), threaten native ecosystems, biodiversity, and habitats. Mauritius’ native biodiversity is already critically endangered, with 100 extinctions attributed to past IAS introductions. Ongoing threats to remaining native species such as unique, rare, and threatened animals and plants necessitate minimizing the impact of IAS [108].

The present study highlights the invasive potential of several *Ipomoea* species previously overlooked. Presently, *I. alba* is observed to exhibit aggressive invasion through easy dispersal of fragments and seeds in waterways and form extensive monocultures at riparian zones, forest lands, and gorges, smothering native vegetation and transforming the natural habitat. *I. alba* is recognized by the Global Compendium of Weeds as an invasive weed in various regions, including China, South Africa, Cuba, and island chains like Australia, New Zealand, and Hawaii in the Pacific [109]. Similarly, *I. indica* exhibited dominance in several areas by outcompeting tall trees and shrubs. This invasive behavior has been widely documented, in sources like CABI Compendium [110], PIER [111], Weeds of New Zealand [112], and the Queensland Government [113], all reporting similar detrimental effects. *I. pes-caprae*, on the other side, presented a more nuanced case. While demonstrating invasive tendencies through lateral spread on shorelines, its role in restoring and stabilizing dunes [114,115] necessitates population control measures that balance erosion mitigation with potential ecological disruption. Further studies are required to definitively assess its threat to coastal ecosystems. *I. tilliacea* populations in humid habitats exhibit concerning invasive potential, emulating the aggressive behavior of *I. alba* by occupying riparian areas, and gorges and extending vines deep into existing vegetation. *I. cairica*’s invasiveness ranges from moderate to severe, potentially engulfing entire support structures if left uncontrolled. *I. ochracea* can sometimes show some degree of invasiveness in open habitats and free spaces by climbing and forming dispersed canopies. Unlike other *Ipomoea* species, populations of *I. obscura* growing on fences do not display substantial invasive behavior but may rather appear as an eyesore. However, they may contribute to the overall invasiveness of pre-established herbs in open areas. These observations set an alarm for considering concrete actions to control the invasive *Ipomoea* species mentioned above in the management plans like the National Invasive Alien Species Strategy (NIASS), National Invasive Alien Species Strategy and Action Plan (NIASSAP), or the recent GEF-UNDP project [108] for the Republic of Mauritius. Future steps at selected localities might be needed to cover systematic measurements of the population size using appropriate techniques such as belt transects, quadrats, or line transects [116] to monitor the status of the species population and determine the need for management and as well as conservation.

### 4.3. Conservation

Mauritius, a globally recognized biodiversity hotspot as designated by the IUCN in 1993 [117], is characterized by a unique geographical setting and tropical climate that is believed to have fostered the evolutionary adaptation of species [118]. The country’s exceptional levels of endemism, with 39% of plants, 80% of non-marine birds, 80% of reptiles, and 40% of bat species found exclusively on the island, underscore its critical role in global biodiversity conservation [119]

Though some *Ipomoea* species, such as *Ipomoea batatas* (sweetpotato) [31,120,121] and *I. aquatica* [121,122], are valued, several *Ipomoea* species are considered weedy invasive plants [49,52,53,54] and agricultural pests [31]. In Mauritius, 17 *Ipomoea* species are alien and have been naturalized to varying degrees. *Ipomoea pes-caprae* subsp. *brasiliensis* and *I. violacea* are considered indigenous to the Mascarene Islands [32].

Nevertheless, due to the isolation and remoteness of the island from the countries of origin of *Ipomoea*, limited gene flow may have given rise to a unique genetic diversity of the *Ipomoea* in forms of ecotypes that deserve to be conserved for their characterization and evaluation of beneficial traits for sweetpotato improvement programs. Presently, the National Field Gene Bank has no accession to wild relatives of agricultural crops, including sweetpotato, and it is suggested that actions be taken to collect samples across the wild populations for conservation in the field gene bank.

The International Union for Conservation of Nature’s Red List of Threatened Species [66] shows that globally some *Ipomoea* species (*I. cairica*, *I. triloba*, *I. littoralis*, *I. ochracea*, *I. alba*, *I. pes-caprae*, *I. aquatica*, *I. tiliacea*) have been listed as Least Concern (LC). While most of the *Ipomoea* species are abundant in Mauritius, several species including *I. cairica* with white flower, *I. nil*, *I. purpurea*, *I. carnea*, *I. herderifolia,* and *I. quamoclit* were found in only a few localities and could be under threat. These species were thriving in regions susceptible to human activities such as urban development, residential construction, use of herbicides in weed control, and agricultural expansion, and are under threat. During our surveys, we experienced the disappearance of the only population of *I. triloba* in Réduit, (one of three sites that they encountered in Mauritius) due to management of the roadside verges with the use of herbicides.

With the increasing severity of droughts and floods in Mauritius, the island is considered to have one of the most vulnerable endangered island floras in the world [117,123,124]. With heavy deforestation for agriculture and urban development, natural forests have been degraded. Only around 2% of the land area of Mauritius is considered to be covered with good-quality native forests [125]. If this vast genetic pool, occurring on the basis of the island’s location, age, isolation, and varied topography [123], remains unexplored, it is evident that the persistent climatic instability, assault of increasing threats, including land-use change, pests, and diseases, will cause remarkable genetic erosion of these CWRs and landraces.

It is suggested that a number of complementary conservation actions be taken to safeguard the genetic diversity of the wild *Ipomoea* species in Mauritius. The National Strategic Action Plan for the conservation and sustainable use of crop wild relatives in The Republic of Mauritius [126] provides a framework for their conservation. This National Strategy prepared under an EU-ACP project identified 527 CWR species on the Mauritius mainland and 142 species on Rodrigues Island, including those of *Ipomoea* species. Although CWRs of sweetpotato were not prioritized for conservation actions in Mauritius, those on Rodrigues Island were. The NSAP for CWRs reviewed the conservation status of CWRs in the republic both in the wild (in situ) and in ex situ conservation facilities and recommended a set of concrete conservation actions, including setting up CWR genetic reserves, ecogeographic studies, control of invasive species, an extension of protected areas system to include CWRs, and in situ monitoring of wild populations. Further complementary action to collect and back up samples of the wild population in an ex situ gene bank and duplicates in other gene banks abroad, as safety duplication, was recommended. It is considered that an in situ conservation approach allows the evolution and adaptation of species with regard to new variations like gradual changes in environmental conditions and biotic interactions while preserving the current biodiversity [8,127]. Nevertheless, to ensure the conservation of the reservoir of genetic diversity, the in situ conservation approach needs to be complemented by the ex situ collections and to make them more accessible for use [8]. Currently, none of the wild relatives of sweetpotato is conserved ex situ. A priority would be to collect the seeds (if they are orthodox to conserve the National Gene Bank or else collect planting material to conserve in the field gene bank. It is suggested that these conservation approaches be applied as a priority to *Ipomoea* species identified as the most threatened species including *I. cairica* white flower, *I. herderifolia*, *I. quamoclit*, *I. nil*, and *I. pupurea*. To propose the most convenient approach, further investigation is required considering factors like the inter- and intra-crossability of the species, seed storage behavior of the wild species, the needs and constraints of the methods, and, most importantly, the feasibility with regards to the economical aspect.

In conclusion, the distribution pattern of the 16 species of CWRs of sweetpotato was studied across different agro-climatic regions on the mainland of Mauritius. The study revealed that most species prefer humid and super-humid localities and are mainly found in disturbed habitats. It was also observed that several of the *Ipomoea* species are invasive and need to be properly managed and controlled. Given the plasticity of many of the species, it is important that the breadth of the diversity found in the wild populations be conserved in situ to allow them to continue to evolve and adapt to the changing environment and that they are complemented by conservation in the National Gene Bank in Mauritius to enable greater access for research and crop development.

## Figures and Tables

**Table 1:** The respective species number of sites and percentage occurrence following this ecogeographic study.

Species	No of Site	Percentage Occurrence
*I. indica*	137	29.0
*I. alba*	123	26.0
*I. obscura*	107	22.6
*I. cairica*	44	9.3
*I. pes-caprae*	14	3.0
*I. tilliacea*	12	2.5
*I. ochracea*	7	1.5
*I. batatas*	6	1.3
*I. carnea*	5	1.1
*I. nil*	5	1.1
*I. hederifolia*	4	0.6
*I. triloba*	3	0.6
*I. quamoclit*	2	0.4
*I. littoralis*	2	0.4
*I. aquatica*	2	0.4
*I. purpurea*	1	0.2

**Table 2:** Total number of Ipomoea individuals encountered per district and the district’s respective richness, diversity, and evenness indices.

Summary Statistics:				
Variable	Richness	Total Number of Individuals	Shannon Diversity Index	Pielou Evenness Index
Moka	9	91	1.85	0.84
Pamplemousses	9	63	1.52	0.69
Black River	8	30	1.73	0.83
Grand Port	8	75	1.33	0.64
Plaines-Wilhems	7	93	1.32	0.68
Savanne	7	58	1.62	0.83
Flacq	7	28	1.51	0.77
Rivière du Rempart	7	26	1.32	0.68
Port-Louis	1	3	0	-

**Table 3:** The areas delimitated per district, for the study, to be representative of Mauritius island climatology and topography.

Areas Delimitated within the Districts of Mauritius			
Pamplemousses	Riviere du Rempart	Flacq	Grand Port
Terre Rouge	Grand Baie	St Julien d’Hotman	Grand sable
Tombeau Bay	Vale	Bon Accueil	BambousVirieux
Balaclava	Pereybere	Lalmatie	Bois des Amourettes
Pointe aux Piments	Petit Raffray	Queen Victoria	Vieux Grand Port
Pointe aux Biches	Goodlands	Central Flacq	Ferney
Troux aux Biches	Roche noire	Quatre Cocos	Riviere des Creoles
Pamplemousses	Mare d’australia	Belle mare	Mahebourg
Triolet	Rivière du Rempart	Palmar	Plaine Magnien
Ville Bague	Cottage	Trou d’Eau Douce	Trois Boutiques
Mont Goût	Cap Malheureux	Beau champ	Le bouchon
	Poudre d’Or	Grand River South East	Mon Desert
	Calodyne	Quatre Soeurs	L’escalier
		Sebastopol	Mare D’albert
		Belle Rive	Rose Belle
		Belle Air	Banane/clunny
**Savanne**	**Black River**	**Plaine Wilhems**	**Moka**
Grand Bois	Chamarel	Le Pétrin	Réduit
Bois Cheri	Le Morne	Midlands	Verdun
Grand Bassin	La Gaulette	La vigie	Quartier-Militaire
Black River Gorges	Case Noyale	Eau Coulée	Belle Rive
Bassin blanc	Grande Riviere Noire	Camp Fouquereaux	Providence
Chamouny	La Preneuse	Curepipe	Melrose
Chemin Grenier	Tamarin	Trou aux Cerfs	Montagne Blanche
St Felix	Bamboo	Valentina	Alma
Riambel	Gros cailloux	Vacoas-Phoenix	
Pomponette beach	Albion	Mon Désir	
Souillac		Carreau laliane	**Port Louis**
Gris gris		Solferino	Long Mountain
St Aubin		Quatre Bornes	Caroline
Rivière des Anguilles		Ebène	Roche Bois
Britannia			

## Data Availability

Data are available upon request.
